# Regulon active landscape reveals cell development and functional state changes of human primary osteoblasts in vivo

**DOI:** 10.1186/s40246-022-00448-2

**Published:** 2023-02-15

**Authors:** Shengran Wang, Yun Gong, Zun Wang, Xianghe Meng, Zhe Luo, Christopher J. Papasian, Jonathan Greenbaum, Yisu Li, Qilan Liang, Yiping Chen, Xiaohua Li, Qiu Xiang, Hiuxi Zhang, Ying Liu, Liang Cheng, Yihe Hu, Lijun Tan, Hui Shen, Hongmei Xiao, Hongwen Deng

**Affiliations:** 1 Center for System Biology, Data Sciences and Reproductive Health, School of Basic Medical Science, Central South University, Changsha, 410008 China; 2 Tulane Center of Biomedical Informatics and Genomics, Deming Department of Medicine, Tulane University School of Medicine, Tulane University, 1440 Canal St., Suite 1610, New Orleans, LA 70112 USA; 3 Xiangya School of Nursing, Central South University, Changsha, 410013 China; 4 Department of Biomedical Sciences, School of Medicine, University of Missouri-Kansas City, Kansas City, MO USA; 5 Department of Cell and Molecular Biology, Tulane University School of Science and Engineering, Tulane University, New Orleans, LA 70112 USA; 6 Laboratory of Molecular and Statistical Genetics, College of Life Sciences, Human Normal University, Changsha, 410081 China; 7 Department of Orthopaedics and National Clinical Research Center for Geriatric Disorders, Xiangya Hospital, Central South University, Changsha, 410008 China; 8 Department of Orthopedics, Xiangya Hospital, Central South University, Changsha, 410008 China; 9 Institute of Reproductive and Stem Cell Engineering, Center of Reproductive Health, School of Basic Medical Science, Central South University, 172 Tongzipo Road, Yuelu District, Changsha, 410013 Hunan Province People’s Republic of China; 10 Center of Reproductive Health, School of Basic Medical Science, Central South University, Changsha, 410008 China

**Keywords:** Osteoblast, Bone metabolism, Single-cell RNA sequencing, Transcription factor, Cell-specific network

## Abstract

**Background:**

While transcription factor (TF) regulation is known to play an important role in osteoblast development, differentiation, and bone metabolism, the molecular features of TFs in human osteoblasts at the single-cell resolution level have not yet been characterized. Here, we identified modules (regulons) of co-regulated genes by applying single-cell regulatory network inference and clustering to the single-cell RNA sequencing profiles of human osteoblasts. We also performed cell-specific network (CSN) analysis, reconstructed regulon activity-based osteoblast development trajectories, and validated the functions of important regulons both in vivo and in vitro.

**Results:**

We identified four cell clusters: preosteoblast-S1, preosteoblast-S2, intermediate osteoblasts, and mature osteoblasts. CSN analysis results and regulon activity-based osteoblast development trajectories revealed cell development and functional state changes of osteoblasts. *CREM* and *FOSL2* regulons were mainly active in preosteoblast-S1, *FOXC2* regulons were mainly active in intermediate osteoblast, and *RUNX2* and *CREB3L1* regulons were most active in mature osteoblasts.

**Conclusions:**

This is the first study to describe the unique features of human osteoblasts in vivo based on cellular regulon active landscapes. Functional state changes of *CREM, FOSL2, FOXC2, RUNX2*, and *CREB3L1* regulons regarding immunity, cell proliferation, and differentiation identified the important cell stages or subtypes that may be predominantly affected by bone metabolism disorders. These findings may lead to a deeper understanding of the mechanisms underlying bone metabolism and associated diseases.

**Supplementary Information:**

The online version contains supplementary material available at 10.1186/s40246-022-00448-2.

## Introduction

Bone homeostasis is highly dependent on coordinated and sequential actions by osteoblasts, osteoclasts, and osteocytes [1]. Osteoblasts mainly promote bone formation and influence bone resorption in this dynamic and well-balanced regulation [1, 2]. Under induction by a series of key pathways and transcription factors (TFs), osteoblasts reach maturity and actively modify the bone microenvironment [3–7]. For example, runt-related transcription factor 2 (*RUNX2*) is an early marker and master TF for osteoblast differentiation that shows different activation patterns through the osteoblast differentiation process [8–11]. Targeted disruption of *RUNX2* results in a complete lack of bone formation, owing to maturational arrest of osteoblasts [12]. Thus, exploring the dynamic changes of gene function and TF activation, from early to mature stages of osteoblast differentiation, is important in understanding the pathophysiology of bone homeostasis and related bone loss disease.

Previous studies have provided evidence for heterogeneity among osteoblasts [13, 14], including our previous study which provided the first unbiased examination of the cellular landscape of freshly isolated human osteoblasts via single-cell RNA sequencing (scRNA-seq) [15]. We identified cell heterogeneity among human osteoblasts in vivo, and preliminarily explored transcriptome dynamic changes based on gene expression. Accumulating evidence suggested that many pathophysiological processes are not only controlled by the expression states of one or several molecules but rather by coordinated expression changes of a number of genes [16–19]. Network analysis, accounting for expressional changes of multiple genes simultaneously, provides an opportunity to explore specific biological processes more comprehensively, under conditions resembling their natural biological state. However, no research has explored osteogenic processes based on changes in TF networks and gene interactions at the single-cell level.

Single-cell regulatory network inference and clustering (SCENIC) is a method of performing simultaneous reconstruction of gene regulatory networks, permitting identification of regulons and cell states [20]. Regulons are composed of a TF as regulatory gene and the target genes of the TF. The regulatory gene generally regulates the regulon as a unit. Another approach is to construct a cell-specific network (CSN), separate gene regulatory networks for individual cells, which allows for identification of cell type heterogeneity based on multiple genes and their co-expressions [16]. TF networks and CSN analysis have been efficiently applied to provide potentially impactful insights regarding embryonic development, aging processes, and tumorigenesis [16–19].

TF abnormalities are known to be related to some common skeletal diseases [21–23]. For example, clinical research has revealed associations between Osx and age-related osteoporosis [22]. In the present study, we performed TF network and CSN analysis in human osteoblasts for the first time, with the objective of exploring their biological function in bone-related physiological processes. Our work revealed five important regulons and permitted reconstruction of osteoblast development trajectories based on regulon activity. Our findings provide a framework for understanding gene relationships during osteogenesis at the single-cell level, thereby laying the foundation for exploring characteristic gene functions from a novel TF regulation perspective.

## Materials and methods

### Single-cell RNA sequencing (scRNA-seq) data information

As described in our recent study [15], the study subject was a 31-year-old male patient who suffered from osteoarthritis and osteopenia (BMD T-score: 0.6 at lumbar vertebrae, − 1.1 at total hip). His cardinal manifestations were hip pain and limited activity/functionality of the hip joint. He underwent hip replacement surgery at the Xiangya Hospital of Central South University to treat osteoarthritis. The subject had bone mineral density (BMD) measurement by dual energy x-ray absorptiometry (DXA) prior to surgery and was screened with a detailed questionnaire for medical history and a physical examination to rule out preexisting chronic conditions which may significantly influence bone metabolism, such as diabetes mellitus, renal failure, liver failure, hematologic diseases, disorders of the thyroid/parathyroid, malabsorption syndrome, malignant tumors, and previous pathological fractures [15]. The femoral head was collected from the patient during hip replacement surgery. Freshly harvested bone tissue fragments were incubated with highly purified, endotoxin-free type II collagenase. Collected cells were incubated with human CD31/34/45-PE, human ALPL-APC, and 7-AAD antibody. After negative selection of 7-AAD, ALPL + /CD45/34/31- cells were collected as osteoblasts on the BD FACS Aria II sorter. The scRNA-seq library was constructed using the Single-Cell 3’ Library Gel Bead Kit, version 3 (10X Genomics), and 150 bp paired-end sequencing was performed on Illumina Novaseq6000 platform. Cellular barcodes in raw sequencing data were demultiplexed using Cell Ranger analysis pipeline v3.0. Reads were aligned to human genome version GRCh38 (hg38). Low-quality cells or empty droplets often have very few genes, while cell doublets or multiplets may exhibit an aberrantly high gene number. We set the lower gene number limit (200 genes) to eliminate low-quality cells or empty droplets and the upper limit (5000 genes) to eliminate cell doublets or multiplet droplets (Additional file 1: Fig. S1A) [15, 24]. For osteoblast lineage cell filtering, only cells expressing the gene *RUNX2*, an early marker of osteoblast differentiation, were included for further analysis. Cells with a mitochondrial gene percentage over 15% or expressed hemoglobin genes (*HBM, HBA1, HBA2* and *HBB*) were also discarded. The filtered gene expression matrix containing the remaining 3507 cells was normalized to a total of 10,000 molecules per cell by the NormalizeData function in Seurat R package (v3.6.1) [25].

### Gene expression-based osteoblast lineage cell clustering

The top 2000 highly variable genes selected by the FindVariableFeatures function in Seurat R package were used as inputs for initial principal component analysis (PCA). We then performed a jackstraw analysis [26] to select principal components (PCs) which separated the cells effectively. Afterward, the first 18 PCs at the plateau region of the elbow plot (Additional file 1: Fig. S1B) were used for uniform manifold approximation and projection (UMAP) dimension reduction. The percentage of variance associated with each PC was calculated according to the standard deviations (stdev) in the elbow plot using the formula: stdev**2/sum (stdev**2) * 100. The cumulative variance explained by the 18 selected PCs was 54.70% (Additional file 1: Fig. S1C). Cell clustering results were visualized in a two-dimensional panel using DimPlot function in Seurat R package.

### Single-cell regulatory network inference and clustering (SCENIC) analysis

We calculated regulation modules based on human hg19 transcriptional regulator database (RcisTarget in Bioconductor on hg19-tss-centered-10 kb-7species.mc9nr.feather) using SCENIC R package [20]. Target genes that were co-expressed with transcription factors were identified using GENIE3. Regulons were identified from co-expression and DNA motif analyses. Area under the curve (AUC) scores were calculated to evaluate regulon activity as a whole (as opposed to the TF or individual genes alone) in each cell using AUCell function. This approach is robust against drop-out events of individual genes and provides a unique perspective to explore cell states. The output matrix, with the AUC score for each regulon in each cell, was used directly as continuous input to perform cell clustering and trajectory inference. The regulon regulatory network was visualized in Cytoscape (v3.6.1), and the Kruskal–Wallis test was used for multi-group expression comparison (Student’s *t* test is not appropriate in this study because the data under analysis do not follow normal distributions).

### Constructing single-cell trajectories in osteoblast

To discover developmental transitions, single-cell developmental trajectories were constructed in Monocle 2 R package (v2.14.0) [27]. We used highly variable features to sort cells into pseudotime order with “orderCells” function. “DDRTree” and “UMAP” were applied to reduce dimensional space and their results were used to perform cell trajectory visualization. Trajectory plots were generated by the “plot_cell_trajectory” function, while dendrograms were generated by “plot_complex_cell_trajectory” function in Monocle 2.

Diffusion mapping is another method for dimensionality reduction that is often used to identify bifurcation and pseudotimes. Our analysis was conducted using the R Bioconductor destiny package (v3.0.1) [28] with default parameters. The average cell-to-cell transition probabilities between cell types were calculated using destiny and presented in a heatmap. It should be noted that gene expression matrix and AUC score matrix served as the input values to construct gene expression-based and regulon activity-based osteoblast trajectories, respectively.

### Gene set enrichment analysis (GSEA)

To identify the significantly enriched pathways of target gene sets in given clusters, we used GSEA (clusterProfiler R package) to perform enrichment analysis. Only terms showing false discovery rate (FDR) adjusted *p* values (BH) less than 0.05 and absolute value of normalized enrichment score (NES) greater than 1 were considered significantly enriched.

### Protein–protein interaction (PPI) network

We used the Search Tool for the Retrieval of Interacting Genes (STRING) database to build PPI networks in selected target genes. Then, molecular complex detection (MCODE) plugin was used to identify the most densely connected core network modules in the PPI network. MCODE is a graph theoretic clustering algorithm based on vertex weighting. Local neighborhood density and outward traversal from a locally dense seed protein are analyzed to isolate the dense regions in the PPI network. Key parameters were set as default as follows: degree cutoff = 2, node score cutoff = 0.2, K-core value = 2, and MCODE score cutoff value = 3.0 by Cytoscape (v3.6.1) [29].

### Cell-specific network (CSN) analysis

**Fig. 1:**
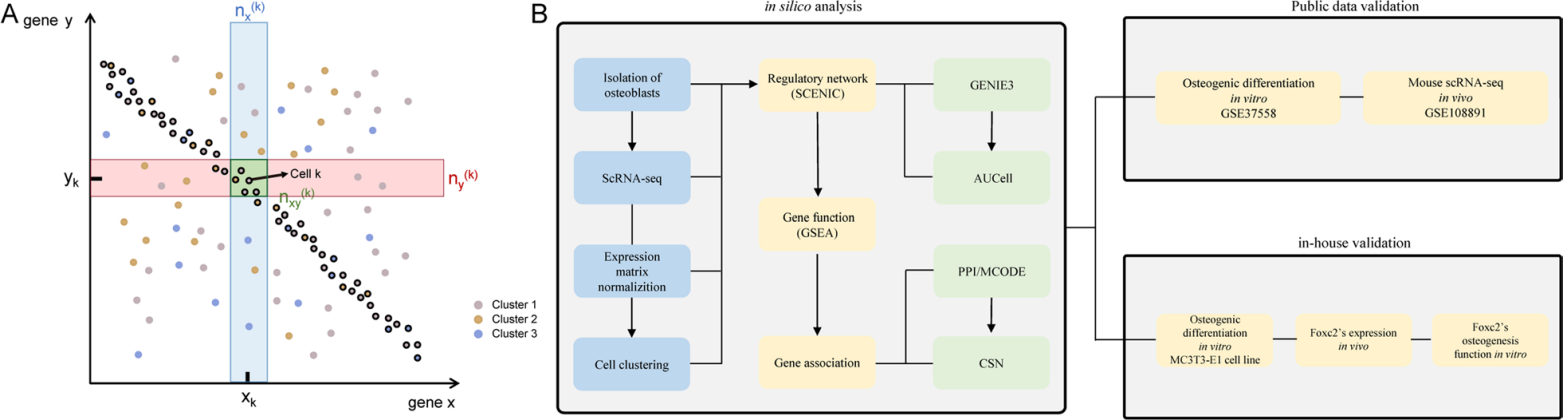
Flowchart and scatter diagram for gene *x* and gene *y*. **A** Each dot in the scatter diagram represents an individual cell. *X*-axis represents the expression values of gene *x*, *Y*-axis represents the expression values of gene *y*. The green dot represents cell *k*. The number of dots in the blue, red, and intersection green boxes near the green dot (cell *k*) is denoted as *n*_*x*_^(*k*)^, *n*_*y*_^(*k*)^ and *n*_*xy*_^(*k*)^, respectively. Colored by cell cluster. Dots with black outlines represent cells with significant inter-relationship edge of gene *X*–*Y*. **B** Workflow of the research

We performed CSN analysis in MATLAB software as previously described [16]. Briefly, a scatter plot is constructed based on the expression values of genes *x* and *y* in different cells (Fig. 1A). Each dot represents an individual cell (*X*-axis and *Y*-axis show the expression values of gene *X* and *Y* for cell *k*, respectively), and *n* represents the total cell number in the scatter diagram. The number of dots (i.e., cell number) in the blue, red, and intersection green boxes is denoted as *n* *x*(*k*), *n* *y*(*k*) and *n* *xy*(*k*), respectively. We set *n* *x*(*k*) = *n* *y*(*k*) = 0.1*n* as default. The coefficient 0.1 denotes the box size (blue and red box). In cell *k*, the statistic $\hat{\rho }_{xy}^{{ ^{\left( k \right)} }}$ is used to assess the inter-relationships (edges) between gene *x* and gene *y* (Eq. 1).

1 $$\hat{\rho }_{xy}^{{ ^{\left( k \right)} }} = \frac{{\sqrt {n - 1} \cdot \left( {n \cdot n_{xy}^{{ ^{\left( k \right)} }} - n_{x}^{{ ^{\left( k \right)} }} n_{y}^{{ ^{\left( k \right)} }} } \right)}}{{\sqrt {n_{x}^{{ ^{\left( k \right)} }} n_{y}^{{ ^{\left( k \right)} }} \left( {n - n_{x}^{{ ^{\left( k \right)} }} } \right)\left( {n - n_{y}^{{ ^{\left( k \right)} }} } \right)} }}$$

After hypothesis testing to determine the significance of each edge, the total number of significant edges including gene *x* was returned as network degree matrix (NDM) value for gene *x*. We used the Wilcoxon rank-sum test to identify genes with different NDM values in a given cell cluster compared with others, and adjusted *p* value < 0.05 was regarded as statistically significant.

### Public data for independent in vitro and in vivo validation

The gene expression profile of the osteogenic differentiation process from human bone marrow-derived mesenchymal stem cells (BM-MSCs) to osteoblasts in vitro was obtained from the GEO database with accession number GSE37558 [30]. Total RNA was extracted from cultured BM-MSCs in osteogenic differentiation medium supplemented with 1.8 mM Ca^2+^ for 0, 2, 8, 12, or 25 days. Three replicates were performed for each timepoint, with exception of day 0 which included four replicates. The data were log2 transformed and normalized using “scale” function in R software.

We added another scRNA-seq dataset in vivo on mouse osteoblasts which acquired from GEO database under the accession number GSE108891 [31] to further validate our osteogenic differentiation pseudotime results. This scRNA-seq dataset of mouse osteoblasts used lineage-specific Cre-transgenic mouse crossed to a knock-in reporter strain Col2.3-cre to trace osteoblast cells. After acquiring the expression matrix of the osteoblasts in mouse, we used Monocle 2 to perform the pseudotime analysis.

### Cell lines and reagents

We further performed in-house validation experiments for the key findings of this study. The preosteoblastic cell line MC3T3-E1 was purchased from the American type culture collection (ATCC, USA) and was cultured in α-minimal essential medium (α-MEM; Gibco, Thermo Fisher Scientific, United States) with 10% fetal bovine serum (FBS) and 1% penicillin–streptomycin (Gibco, Thermo Fisher Scientific, United States) solution. Next, the cells were rinsed with PBS and the medium was replaced with osteogenic differentiation medium (MesenCult™ Osteogenic Stimulatory Kit, Stemcell). Cells were harvested at 0, 2, 3, 7, and 14 days after induction.

### Mineralization assay Alizarin Red S staining

Patterns of matrix mineralization in MC3T3-E1 cells were evaluated using Alizarin Red S staining at 0 and 14 days to validate the osteogenic induction process. After washing twice with PBS, cells were fixed with 4% paraformaldehyde (PFA) for 30 min and stained with Alizarin Red S. Fluorescence signals were visualized using a microscope at 4 × magnification.

### RNA isolation and quantitative real time (qRT)-polymerase chain reaction (PCR)

Total RNA of MC3T3-E1 cells (ATCC) was extracted using RNeasy Mini Kit (Qiagen, American, USA). To quantify the relative gene expression level, qRT-PCR was performed using the RevertAid First Strand cDNA Synthesis Kit (Thermo Scientific) and Synergy brands (SYBR) mix (Bio-Rad Laboratories-Life Sciences) on the CFX Opus 96 Real-Time PCR system (Bio-Rad) in a total reaction volume of 20 µl for 40 cycles. Gapdh was used as an inner reference. The relative expression levels were compared across groups using one-way ANOVA. The sequences of primers used in qRT-PCR reactions were:

*Crem*

- F: 5′-CTCGACTCTCAAGACACTTCAC-3′
- R: 5′-ACTAGCAGAAGAAGCAACTCG-3′

*Fosl2*

- F: 5′-CTGCAGCTCAGCAATCTCTT-3′
- R: 5′-CAGCCAAGTGTCGGAACC-3′

*Foxc2*

- F: 5′-AACCCAACAGCAAACTTTCCC-3′
- R: 5′-GCGTAGCTCGATAGGGCAG-3′

*Creb3l1*

- F: 5′-CCCCATCATCGTAGAACAGTAG-3′
- R: 5′-CCTTCCTGCATTCTCTTCCG-3′

*Runx2*

- F: 5′-GAATGGCAGCACGCTATIAAATCC-3′
- R: 5′-GCCGCTAGAATICAAAACAGTIGG-3′

*Col*

- F: 5′-GGCTCTAGAGGTGAACGTGG-3′
- R: 5′-CACCAGGGGCACCATTAACT-3′

*Alp*

- F: 5′-AACCCAGACACAAGCATTCC-3′
- R: 5′-GCCTTTGAGGTTTITGGTCA-3′

*Osx*

- F: 5′-CCTCTCGACCCGACTGCAGATC-3′
- R: 5′-AGCTGCAAGCTCTCTGTAACCATGAC-3′

### Experimental animals

Male C57BL/6J mice were purchased from Jackson Laboratory (Bar Harbor, ME, USA). All mice were fed with autoclaved food and housed in pathogen-free conditions. All experimental procedures were approved by the Ethics Committee of Xiangya Hospital of Central South University (Changsha, China).

### Bone sectioning, immunostaining, and confocal imaging

To verify the expression of *Foxc2* in osteoblasts in vivo, immunostaining was used to stain Foxc2 and Alp proteins in frozen sections of mouse femur. Freshly dissected femur from male C57BL/6 wild-type mouse was fixed in 4% paraformaldehyde overnight followed by decalcification in 14% EDTA for 2 weeks. Samples were cut in 5-µm-thick longitudinally oriented sections. Frozen sections were blocked in PBS with 5% bovine serum albumin (BSA) for 1 h and then stained overnight with rabbit-anti-Alp (Novus Biologicals, NBP2-67295, 1:100) and sheep-anti-Foxc2 (R&D AF6989SP, 1:100). Next, samples were incubated with appropriate secondary antibodies, including donkey anti-rabbit Alexa Fluor 647 (Invitrogen) and donkey anti-sheep Alexa Fluor 488 (Invitrogen). Sections were mounted with anti-fade prolong gold (Invitrogen) and images were acquired with a Zeiss LSM780 confocal microscope.

### Transfection of Foxc2 siRNA

To explore *Foxc2*’s function in osteogenesis process, the expression of *Foxc2* was knocked down by small molecule interference siRNA in osteogenic-induced MC3T3-E1 cells. Nonsense siRNA was transfected as a negative control (NC). siRNA-Foxc2 and siRNA-NC were synthesized by Integrated DNA Technologies (IDT, USA). Lipofectamine™2000 was used to transfer interfere siRNA at 0 and 4 days after induction and harvested at 3 days after interference.

### Workflow

We used our previous scRNA-seq data (GSE147390) [15] to perform single-cell regulatory network inference by SCENIC R package [20]. Co-expression and AUC score calculation were analyzed with GENIE3 and AUCell functions in SCENIC, respectively. After regulon regulatory network identification, functions of target genes were analyzed by GSEA. Then, we use PPI and CSN network to explore different kinds of gene associations of functional genes. We used MCODE plugin to identify core networks/hub genes in the PPI network, and used CSN analysis to explore the cell-specific gene associations. Then, we both used public datasets (gene expression profile in osteogenic cell line in vitro and scRNA-seq data of mouse osteoblast in vivo) and performed our own in-house verification experiments from mouse osteoblast in vivo (immunofluorescence on mouse femur) and in vitro (osteogenic induction and function verification in mouse osteogenesis cell line) to support our TF regulatory analysis results (Fig. 1B).

## Results

### Gene expression and transcription factor regulatory network analysis identified four osteoblast subtypes

**Fig. 2:**
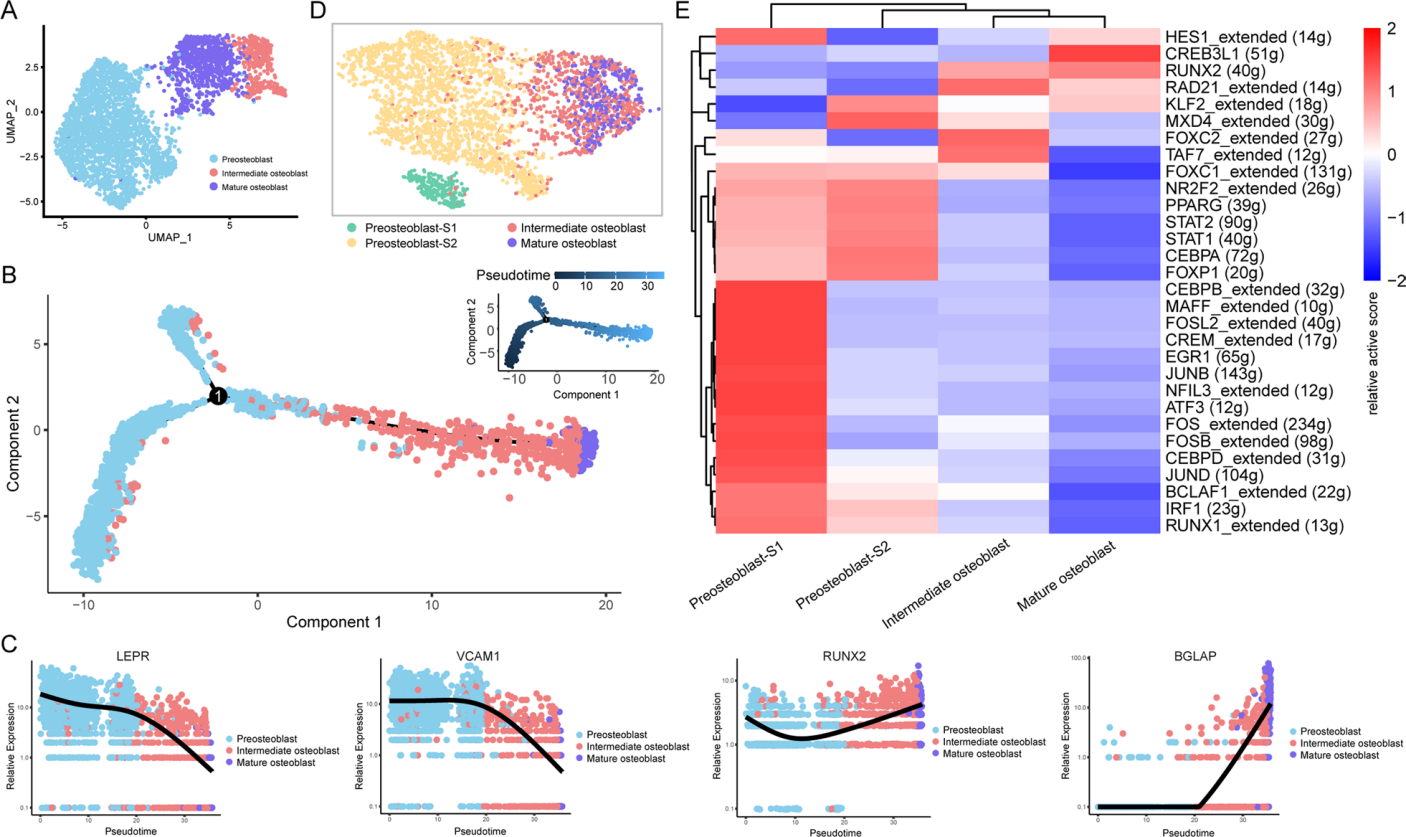
Single-cell clustering and regulatory network inference. **A** Gene expression-based cell clustering results shown using the Seurat clustering layout. Marked out three cell clusters. **B** Cell developmental trajectory inference based on gene expression. Marked three cell clusters. The upper-right trajectory plot indicates the direction of pseudotime. **C** Expression levels (log-normalized) of indicated genes in the three osteoblast clusters with respect to their pseudotime coordinates. The *x*-axis indicates the pseudotime, while the *y*-axis represents the log-normalized gene expression levels. Black lines depict the LOESS regression fit of the normalized expression values. **D** Gene expression and TF regulatory network conjoint clustering results shown using the SCENIC clustering layout. Marked out four cell clusters. **E** Regulons active heatmap in four cell clusters

To understand the molecular features of human osteoblasts, we integrated cell clustering information obtained from gene expression profiles (by Seurat) and regulon activity profiles (by SCENIC). Three cell clusters of osteoblasts were identified based on systematic differences in their gene expression profiles (Fig. 2A). According to their order in the developmental trajectory (Fig. 2B) and their dynamic changes of the expression of BM-MSC and osteoblast-related marker genes (BM-MSC-related markers: *LEPR* [3], *VCAM1* [4], osteoblast-related markers: *RUNX2* [5], *BGLAP* [6]; Fig. 2C), we labeled the early-stage cell cluster with high expression of BM-MSCs-related markers as preosteoblasts, and the late-stage cell cluster with a high expression of osteoblast-related markers as mature osteoblasts. “Intermediate osteoblast” refers to the cell cluster in the middle stage of the developmental trajectory. SCENIC cell clustering results further identified two subtypes of preosteoblast [preosteoblast-S1 and preosteoblast-S2; Fig. 2D]. The regulon active scores of these two cell subtypes differed substantially from each other (Fig. 2E). These results reflect the heterogeneity of two cell subtypes of preosteoblasts, and thus, we identified a total of four cell clusters using integrated cell clustering information based on both gene expression and regulon activity.

### Target gene function of active regulons in preosteoblast-S1

**Fig. 3:**
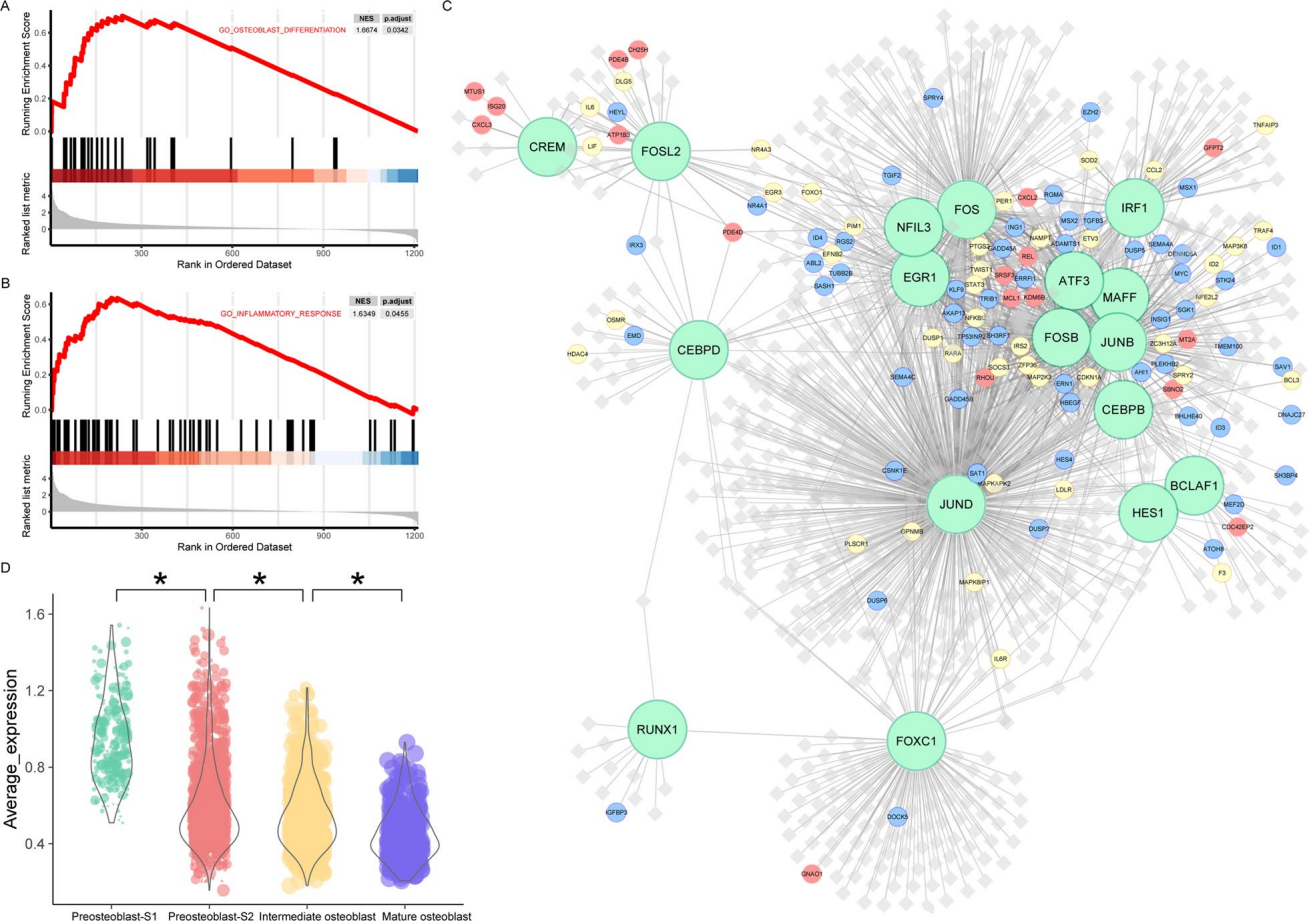
Active regulons in preosteoblast-S1. **A** GSEA analysis results in target genes of active regulons in preosteoblast-S1, “osteoblast differentiation” term. **B** GSEA analysis results in target genes of active regulons in preosteoblast-S1, terms of inflammatory response. **C** Network visualization of active regulons in preosteoblast-S1. Large green dots represent TF, small red dots represent immunity-related target genes, small blue dots represent cell proliferation/differentiation-related target genes, small yellow dots represent both immunity and cell proliferation/differentiation-related target genes; other target genes are shown as gray diamonds. **D** Violin plots showing average expression levels of gene members related to immunity and cell proliferation/differentiation. Dot size represents pseudotime for each cell from early (small) to late (large). *Adjusted *p* value < 0.05 (Kruskal–Wallis test)

According to the transcription factor regulatory network activity heatmap (Fig. 2E), the 17 regulons demonstrating the highest active scores in preosteoblast-S1 were *HES1, FOXC1, CEBPB, MAFF, FOSL2, CREM, EGR1, JUNB, NFIL3, ATF3, FOS, FOSB, CEBPD, JUND, BCLAF1, IRF1* and *RUNX1.* GSEA results showed that gene members in these regulons were mainly enriched in immunity and cell proliferation/differentiation-related function terms such as osteoblast differentiation (Fig. 3A), positive regulation of cell population proliferation, regulation of cell differentiation, regulation of cell population proliferation, regulation of cell activation (Additional file 2: Fig. S2A), inflammatory response (Fig. 3B), response to cytokine, leukocyte migration, cytokine receptor binding, and regulation of lymphocyte activation (Additional file 2: Fig. S2B). We used Cytoscape for network visualization and to mark these categories of gene function (e.g., immunity vs. cell proliferation/differentiation; Fig. 3C). The average expression levels of 113 gene members related to immunity and cell proliferation/differentiation showed a gradual downward trend in the four cell clusters as they matured (Fig. 3D).

### Dynamic changes of *CREM* and *FOSL2* regulon activity

**Fig. 4:**
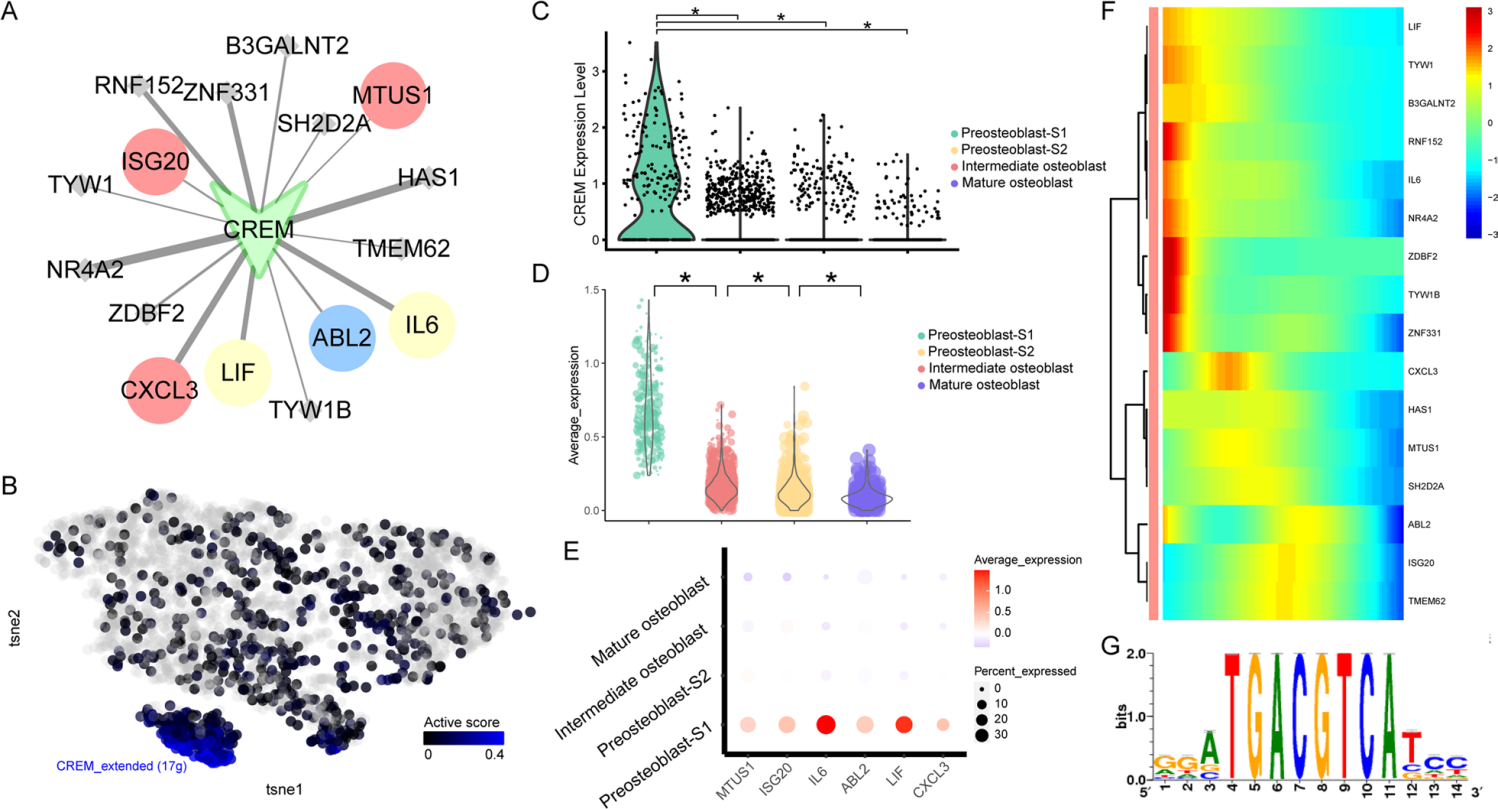
Dynamic changes of *CREM* regulon activity. **A** *CREM* regulatory network. Red dots represent immunity-related target genes, blue dots represent cell proliferation/differentiation-related target genes, yellow dots represent both immunity and cell proliferation/differentiation-related target genes; other target genes are shown as gray dots. **B** *CREM* regulon activity, embedded on SCENIC clustering layout and colored by *CREM* regulon active levels. Gray dots represent completely inactive cells with zero active scores. Other dot colors represent active score in each cell from black (low) to blue (high). **C** Violin plots showing expression levels of *CREM*. *Adjusted *p* value < 0.05 (Kruskal–Wallis test). **D** Violin plots showing average expression levels of all target genes in *CREM* regulon. *Adjusted *p* value < 0.05 (Kruskal–Wallis test). **E** Immunity and cell proliferation/differentiation-related target gene expression in *CREM* regulon among four cell clusters. Dot color indicates the relative expression levels and the dot size shows the proportion of cells expressing the indicated genes. **F** Continuum of dynamic target gene expression in pseudotime of osteoblasts. Pixel color indicates the expression levels. Early-stage cells are list in on the left. **G** Motif taipale_cyt_meth_ATF4_GGATGACGTCATCC_eDBD of *CREM*. The relative sizes of the letters indicate the frequency of four bases in the sequences

The *CREM* regulon showed the highest active score (Fig. 2E) in preosteoblast-S1. There were 16 target genes in this regulon. Among these genes, *ISG20, CXCL3, LIF, ABL2, IL6,* and *MTUS1* were related to immunity and/or cell proliferation/differentiation (Fig. 4A). Both *CREM* regulon activity and *CREM* gene expression showed specific high levels in preosteoblast-S1 (Fig. 4B, C). The average expression for target genes and expression levels for *ISG20, CXCL3, LIF, ABL2, IL6,* and *MTUS1* were reduced gradually among the four cell clusters (preosteoblast-S1, preosteoblast-S2, intermediate osteoblast, and mature osteoblast, Fig. 4D, E). Target genes of the *CREM* regulon showed high expression levels at the early stage in pseudotime (Fig. 4F). The binding motif for most target genes of *CREM* is Taipale_cyt_meth_ATF4_GGATGACGTCATCC_eDBD (Fig. 4G, Table 1).

**Table 1:** Motif information in *CREM, FOSL2*, *FOXC2, RUNX2,* and *CREB3L1* regulons

TF	Target gene	Motif	nMotifs
*CREM*	*IL6*	transfac_pro__M04746	6
*CREM*	*CXCL3*	elemento__TGACATCA	4
*CREM*	*LIF*	elemento__TGACATCA	8
*CREM*	*ISG20*	transfac_pro__M00801	2
*CREM*	*ABL2*	transfac_public__M00178	2
*CREM*	*MTUS1*	transfac_public__M00178	2
*FOSL2*	*ATP1B3*	cisbp__M3083	28
*FOSL2*	*CH25H*	cisbp__M3083	19
*FOSL2*	*EGR3*	cisbp__M3083	34
*FOSL2*	*HEYL*	cisbp__M3083	7
*FOSL2*	*IL6*	cisbp__M3083	37
*FOSL2*	*IRX3*	cisbp__M3083	13
*FOSL2*	*LIF*	cisbp__M3083	23
*FOSL2*	*NR4A1*	cisbp__M3083	8
*FOSL2*	*NR4A3*	cisbp__M3083	50
*FOSL2*	*PDE4B*	cisbp__M3083	27
*FOSL2*	*PDE4D*	cisbp__M3083	35
*FOSL2*	*PIM1*	cisbp__M3083	6
*FOSL2*	*RGS2*	cisbp__M3083	17
*FOSL2*	*DLG5*	swissregulon__hs__ATF5_CREB3.p2	3
*FOXC2*	EFNA1	hocomoco__FOXL2_MOUSE.H11MO.0.C	4
*FOXC2*	SMAD7	hocomoco__FOXL2_MOUSE.H11MO.0.C	5
*FOXC2*	SNAI1	hocomoco__FOXL2_MOUSE.H11MO.0.C	1
*FOXC2*	HEY2	cisbp__M0746	1
*FOXC2*	JAG1	cisbp__M0746	1
*FOXC2*	SEMA7A	cisbp__M0746	1
*FOXC2*	WNT4	cisbp__M0746	1
*RUNX2*	*MEF2C*	transfac_pro__M00984	2
*RUNX2*	*MEPE*	transfac_pro__M00984	2
*RUNX2*	*PDGFC*	transfac_pro__M00984	2
*RUNX2*	*VDR*	transfac_pro__M00984	1
*CREB3L1*	*COL1A1*	hocomoco__CR3L1_HUMAN.H11MO.0.D	29
*CREB3L1*	*SPNS2*	hocomoco__CR3L1_HUMAN.H11MO.0.D	15
*CREB3L1*	*PHOSPHO1*	taipale_cyt_meth__CREB3L1_TGCCACRTCAYN_eDBD_meth	1

**Fig. 5:**
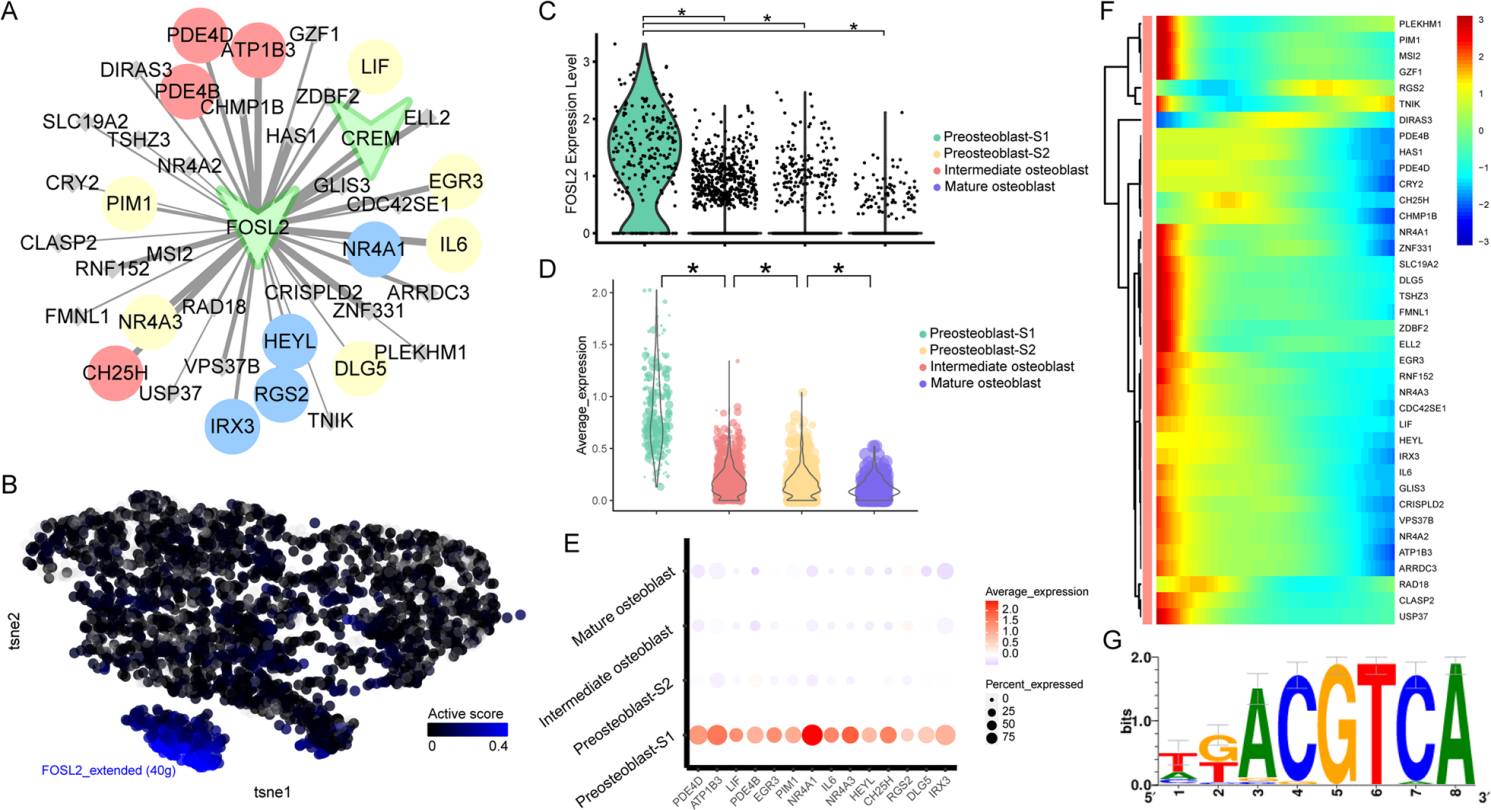
Dynamic changes of *FOSL2* regulon activity. **A** *FOSL2* regulatory network. Red dots represent immunity-related target genes, blue dots represent cell proliferation/differentiation-related target genes, yellow dots represent both immunity and cell proliferation/differentiation-related target genes; other target genes are shown as gray dots. **B** *FOSL2* regulon activity, embedded on SCENIC clustering layout and colored by *FOSL2* regulon active levels. Gray dots represent completely inactive cells with zero active scores. Other dot colors represent active score in each cell from black (low) to blue (high). **C** Violin plots showing expression levels of *FOSL2*. *Adjusted *p* value < 0.05 (Kruskal–Wallis test). **D** Violin plots showing average expression levels of all target genes in *FOSL2* regulon. *Adjusted *p* value < 0.05 (Kruskal–Wallis test). **E** Immunity and cell proliferation/differentiation-related target genes expression in *FOSL2* regulon among four cell clusters. Dot color indicates the relative expression levels and dot size shows the proportion of cells expressing the indicated genes. **F** Continuum of dynamic target gene expression in pseudotime of osteoblasts. Pixel color indicates expression levels. Early-stage cells are listed on the left. **G** Motif cisbp_M3083 of *FOSL2*. The relative sizes of the letters indicate the frequency of four bases in the sequences

*FOSL2*, which was found to be an upstream regulation gene of *CREM,* was the second most active regulon (Fig. 2E) in preosteoblast-S1. 14 of 39 *FOSL2* target genes were related to immunity and/or cell proliferation/differentiation (Fig. 5A). Both *FOSL2* regulon activity and *FOSL2* gene expression showed specific high levels in preosteoblast-S1 (Fig. 5B, C). Average expression for *FOSL2*’s target genes decreased gradually over pseudotime (Fig. 5D). 14 immunity and/or cell proliferation/differentiation-related genes were highly expressed in preosteoblast-S1 (Fig. 5E). Target genes of the *FOSL2* regulon also showed high expression levels at the early stage in pseudotime (Fig. 5F). The binding motif for most target genes of *FOSL2* is cisbp_M3083 (Fig. 5G, Table 1). These collective results demonstrate that the *CREM* regulon, *FOSL2* regulon, and their target genes related to immunity and/or cell proliferation/differentiation were highly active in the preosteoblast-S1 cluster.

**Fig. 6:**
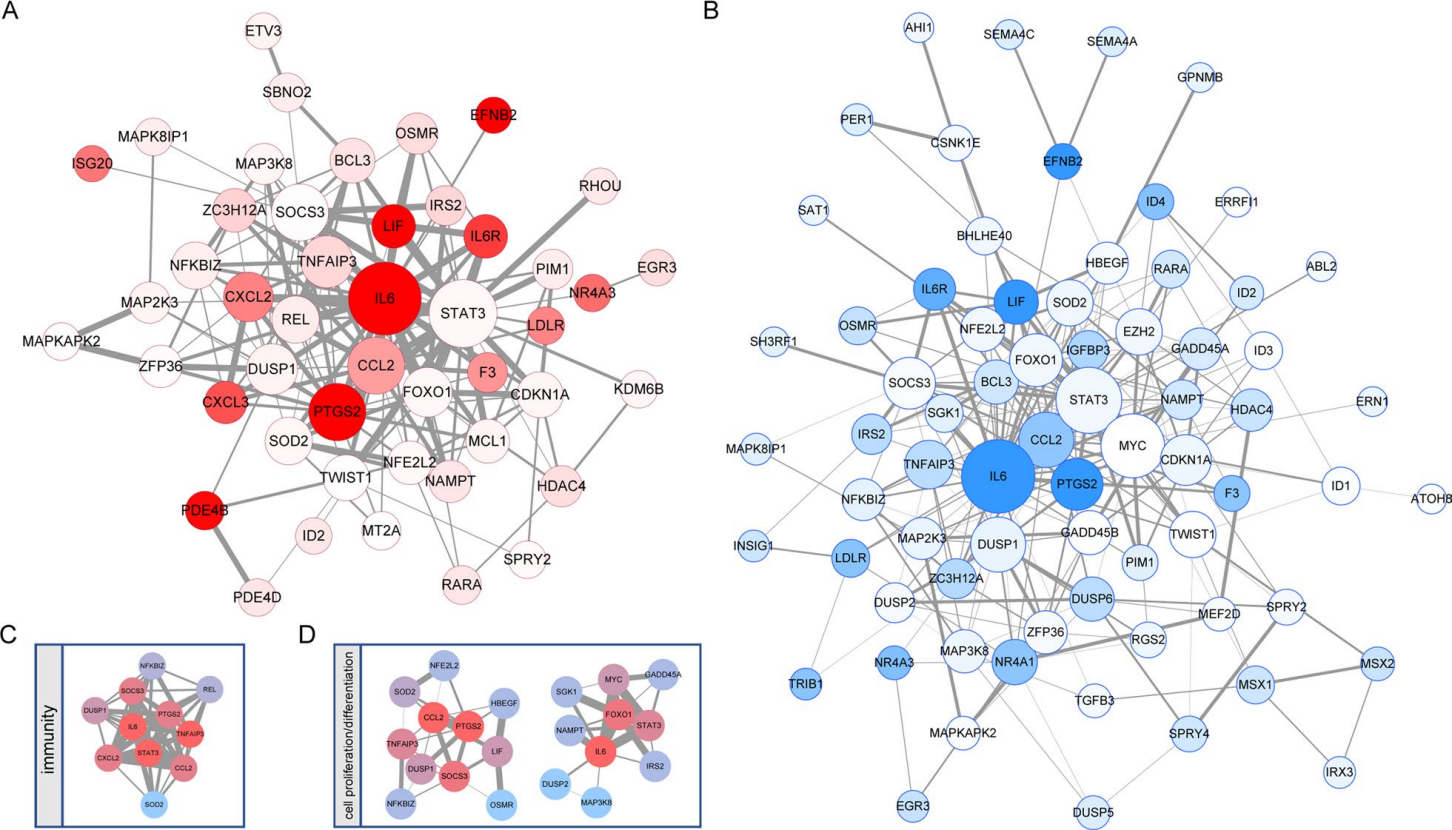
PPI analysis of immunity and cell proliferation/differentiation-related target genes. **A** PPI network of immunity-related target genes. Dot size represents connection degrees from small (low) to large (high). Red nodes have the higher gene expression ratio of pre-osteoblast S1 in comparison with mature osteoblast. Edge width represents the combinded scores. **B** PPI network of cell proliferation/differentiation-related target genes. Dot size represents connection degrees from small (low) to large (high). Blue nodes have the higher gene expression ratio of pre-osteoblast S1 in comparison with mature osteoblast. Edge width represents the combinded scores. **C** Subnetwork in immunity-related PPI network screened by MCODE, MCODE score = 0.94. **D** Subnetwork in cell proliferation/differentiation-related PPI network screened by MCODE, MCODE score = 0.54 (left) and 0.47 (right). Dot color represents connection degrees in each subnetwork from blue (low) to red (high). Edge width represents the combined scores

To identify core target genes in the 17 highly active regulons we identified in preosteoblast-S1, we used immunity and cell proliferation/differentiation-related target genes to construct PPI networks (Fig. 6A, B). Interactions in these networks included correlation/regulation relationships or protein binding validated in Co-IP or other experiments [32]. Combined scores were used to value the confidence of interactions based on these evidences. High combined score interactions showed in wide edges were more valid than other interactions. Larger nodes with the higher network degrees represent the widely association of the corresponding proteins. Node color showed the gene expression ratio of preosteoblast S1 in comparison with mature osteoblast. Genes of the red/blue node were more highly expressed in preosteoblast S1 compared with other genes. MCODE extracted one core gene module in the immunity network with a MCODE score of 9.4 (Fig. 6C). Two core gene modules were extracted in the cell proliferation/differentiation network with MCODE scores of 5.4 and 4.7, respectively (Fig. 6D). Genes included in these core modules may widely influence the function of the whole network with more connection degrees. IL6 and TNFAIP3 were hub genes with high degrees in immunity network core module. IL6, CCL2, and PTGS2 were hub genes with high degrees in cell proliferation/differentiation network core modules. Only IL6 demonstrated the highest degree values in both immunity and cell proliferation/differentiation networks. In addition, IL6 also has many high combined score interactions and a high expression ratio of preosteoblast S1 compared with mature osteoblasts. These results comprehensively demonstrate that four hub genes, particularly IL6, had extensive interactions among core modules and might be critical for coordinating the function of target genes.

### Dynamic changes of *MXD4 *and *KLF2* regulon activity

**Fig. 7:**
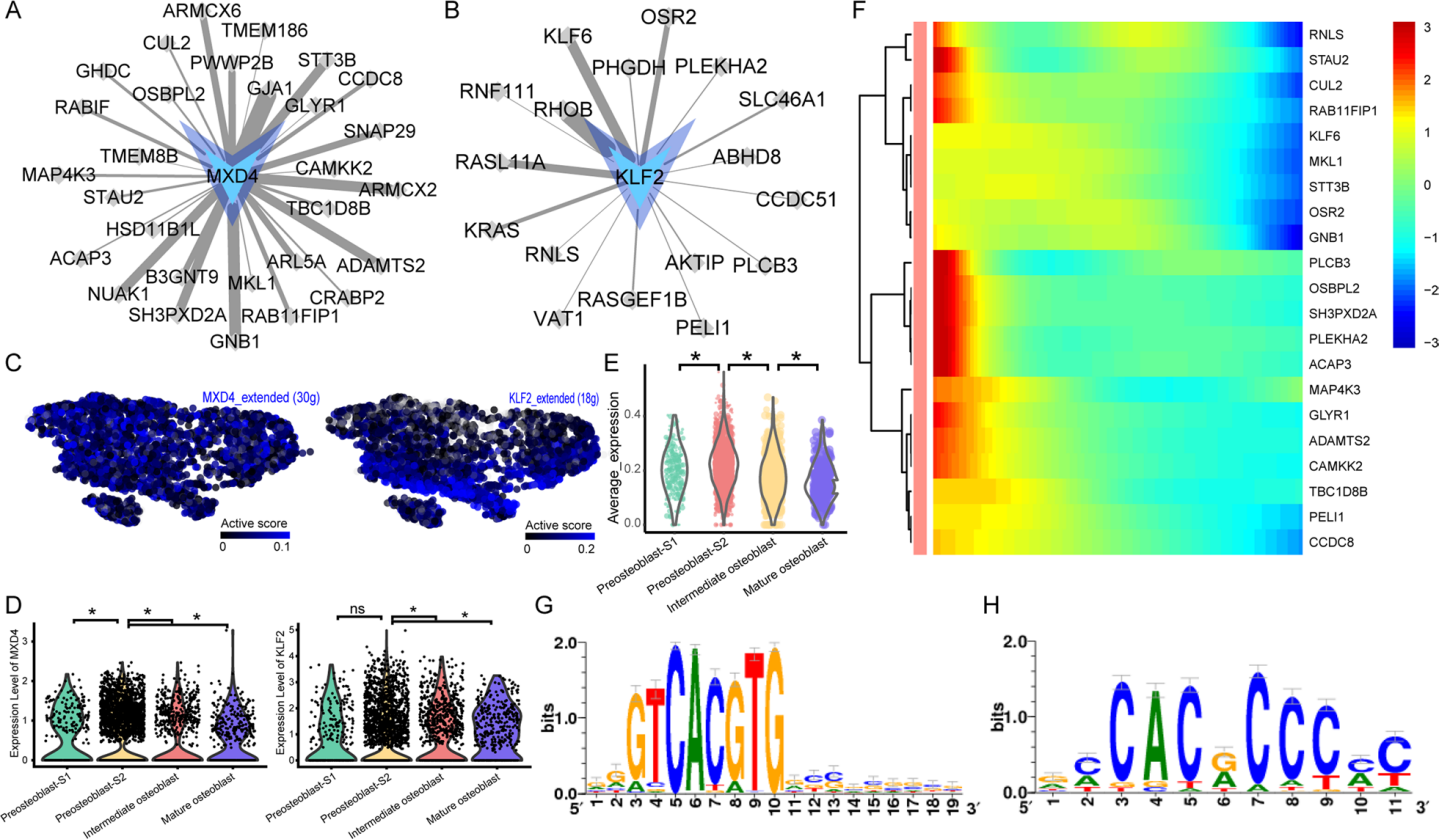
Dynamic changes of *MXD4* and *KLF2* regulons activity. **A** *MXD4* regulatory network. Target genes are shown as gray dots. **B** *KLF2* regulatory network. Target genes are shown as gray dots. **C** *MXD4* and *KLF2* regulon activity, embedded on SCENIC clustering layout and colored by *MXD4* or *KLF2* regulon active levels. Gray dots represent completely inactive cells with zero active scores. Other dot colors represent active scores in each cell from black (low) to blue (high). **D** Violin plots showing expression levels of *MXD4* (left) and *KLF2* (right). *Adjusted *p* value < 0.05 (Kruskal–Wallis test). **E** Violin plots showing average expression levels of all target genes in *MXD4* regulons. *Adjusted *p* value < 0.05 (Kruskal–Wallis test). **F** Continuum of dynamic *MXD4* and *KLF2* regulon’s target gene expression in pseudotime of osteoblasts. Pixel color indicates expression levels. Early-stage cells are list at left. **G** Motif hocomoco__USF2_HUMAN.H11MO.0.A of *MXD4*. **H** Motif transfac_pro__M07913 of *KLF2*. The relative sizes of the letters indicate the frequency of four bases in the sequences

The *MXD4* and *KLF2* regulons showed higher active scores in preosteoblast-S2 (Fig. 2E). There were 29 target genes in the *MXD4* regulon and 17 target genes in the *KLF2* regulon (Fig. 7A, B). Although both *MXD4* and *KLF2* regulon activities showed specific high levels in preosteoblast-S2 (Fig. 7C), *MXD4* gene expression was more specifically higher in the preosteoblast-S2 cell cluster (Fig. 7D). The average expression for target genes in the *MXD4* regulon also showed the highest expression level in preosteoblast-S2 (Fig. 7E). Target gene clusters in the *MXD4* and *KLF2* regulons that showed high expression levels at the early stage in pseudotime are shown in Fig. 7F. The binding motif for most target genes of *MXD4* and *KLF2* is hocomoco__USF2_HUMAN.H11MO.0.A and transfac_pro__M07913 (Fig. 7G, H).

### Dynamic changes of *FOXC2* and *TAF7* regulon activity

**Fig. 8:**
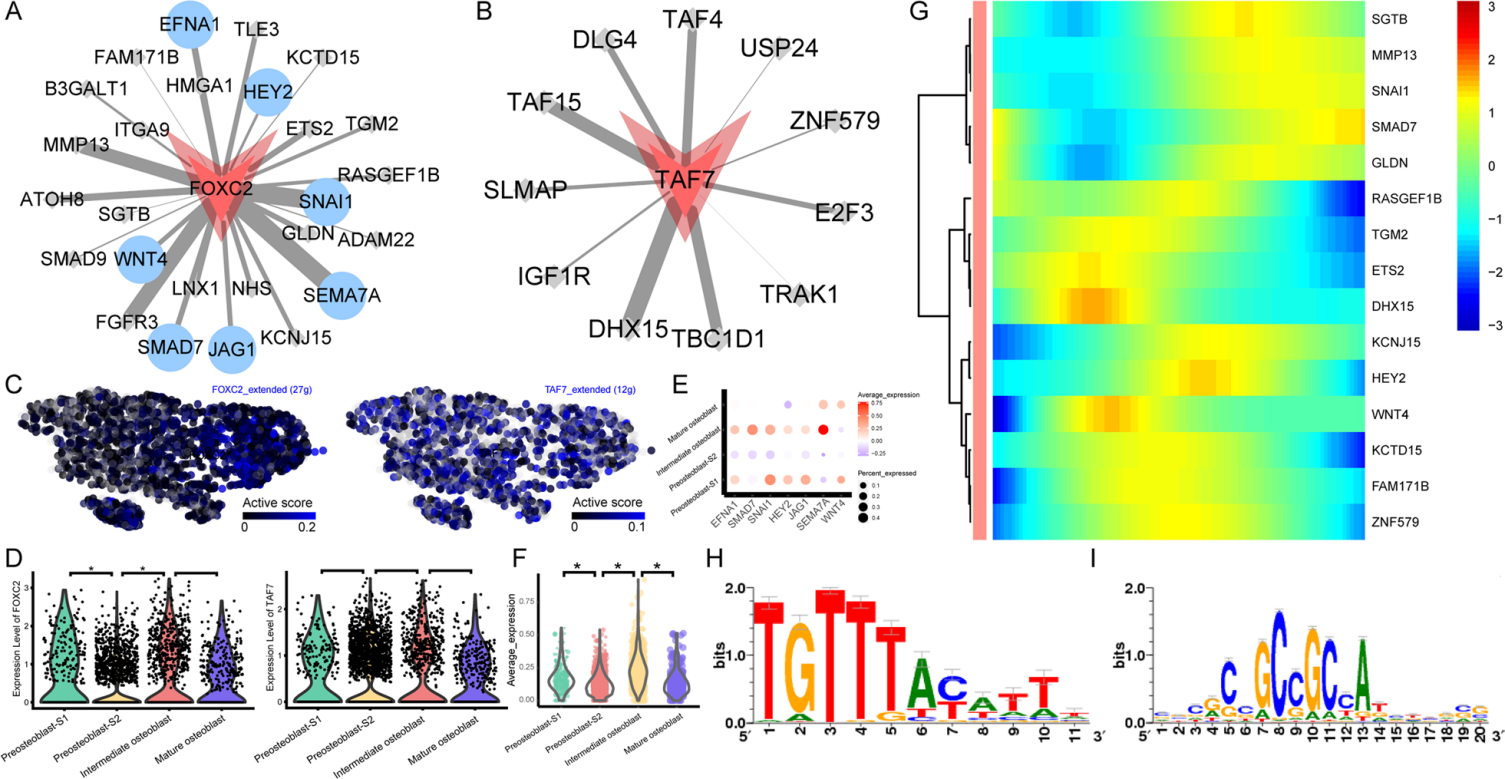
Dynamic changes of *FOXC2* and *TAF7* regulon activity. **A** *FOXC2* regulatory network. Blue dots represent MSC differentiation-related target genes; other target genes are shown as gray dots. **B** *TAF7* regulatory network. Target genes are shown as gray dots. **C** *FOXC2* and *TAF7* regulon activity, embedded on SCENIC clustering layout and colored by *FOXC2* or *TAF7* regulon active levels. Gray dots represent completely inactive cells with zero active scores. Other dot colors represent active scores in each cell from black (low) to blue (high). **D** Violin plots showing expression levels of *FOXC2* (left) and *TAF7* (right). *Adjusted *p* value < 0.05 (Kruskal–Wallis test). **E** MSC differentiation-related target gene expression in *FOXC2* regulon among four cell clusters. Dot color indicates relative expression levels and the dot size shows the proportion of cells expressing the indicated genes. **F** Violin plots showing average expression levels of all target genes in *FOXC2* regulons. *Adjusted *p* value < 0.05 (Kruskal–Wallis test). **G** Continuum of dynamic *FOXC2* and *TAF7* regulon target gene expression in pseudotime of osteoblasts. Pixel color indicates expression levels. Early-stage cells are list at left. **H** Motif hocomoco__FOXL2_MOUSE.H11MO.0.C of *FOXC2*. *I* Motif dbcorrdb__TAF7__ENCSR000BLU_1__m1 of *TAF7*. The relative sizes of the letters indicate the frequency of four bases in the sequences

Active scores for *FOXC2* and *TAF7* regulons were higher in intermediate osteoblasts than they were in the three other cell clusters (Fig. 2E). There were 26 target genes in the *FOXC2* regulon and 11 target genes in the *TAF7* regulon. *EFNA1, SMAD7, SNAI1, HEY2, JAG1, SEMA7A* and *WNT4* in the *FOXC2* regulon are genes related to MSC differentiation in the GO database (Fig. 8A, B). Both *FOXC2* regulon activity and *FOXC2* gene expression showed more specifically higher levels in intermediate osteoblasts than the other stages (Fig. 8C, D). Intermediate osteoblasts showed high expression levels of *EFNA1, SMAD7,* and *SEMA7A* (Fig. 8E) and average expression levels of all *FOXC2* target genes (left plot in 8F). Target gene clusters in the *FOXC2* and *TAF7* regulons that showed high expression levels at the intermediate stage in pseudotime are shown in Fig. 8G. The binding motif for most target genes of *FOXC2* and *TAF7* is hocomoco__FOXL2_MOUSE.H11MO.0.C and dbcorrdb__TAF7__ENCSR000BLU_1__m1 (Fig. 8H, I, Table 1).

### Activity of *RUNX2* and *CREB3L1* regulons increased as osteoblasts matured.

**Fig. 9:**
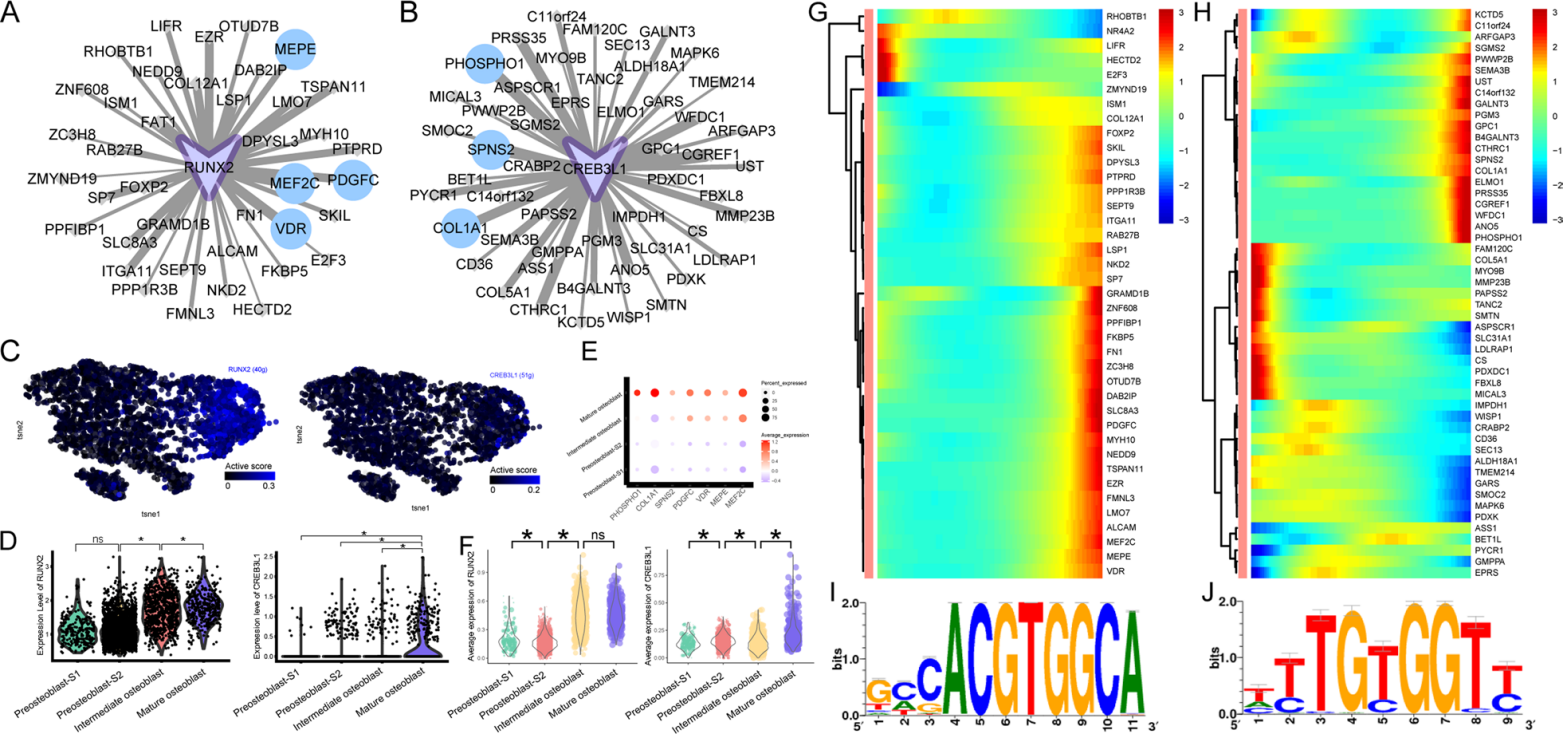
Dynamic changes of *RUNX2* and *CREB3L1* regulon activity. **A** *RUNX2* regulatory network. Blue dots represent skeletal system development-related target genes, other target genes are shown as gray dots. **B** *CREB3L1* regulatory network. Blue dots represent skeletal system development-related target genes, other target genes are shown as gray dots. **C** *RUNX2* and *CREB3L1* regulon activity, embedded on SCENIC clustering layout and colored by *RUNX2* or *CREB3L1* regulon active levels. Gray dots represent completely inactive cells with zero active scores. Other dot colors represent active score in each cell from black (low) to blue (high). **D** Violin plots showing expression levels of *RUNX2* (left) and *CREB3L1* (right). *Adjusted *p* value < 0.05 (Kruskal–Wallis test). **E** Skeletal system development-related target gene expression in *RUNX2* and *CREB3L1* regulon among four cell clusters. Dot color indicates relative expression level and the dot size shows the proportion of cells expressing the indicated genes. **F** Violin plots showing average expression levels of all target genes in *RUNX2* (left) and *CREB3L1* (right) regulons. *Adjusted *p* value < 0.05 (Kruskal–Wallis test). **G** Continuum of dynamic *RUNX2* regulon target gene expression in pseudotime of osteoblasts. Pixel color indicates expression level. Early-stage cells are listed at left. **H** Continuum of dynamic *RUNX2* regulon’s target gene expression in pseudotime of osteoblasts. Pixel color indicates the expression levels. Early-stage cells are listed at left. **I** Motif swissregulon_hs_RUNX1..3.p2 of *RUNX2*. **J** Motif hocomoco_CR3L1_HUMAN.H11MO.0.D of *CREB3L1*. The relative sizes of the letters indicate the frequency of four bases in the sequences

Active scores for *RUNX2* and *CREB3L1* regulons were higher in mature osteoblasts than they were in the three other cell clusters (Fig. 2E). Consequently, we focused on these regulons and performed GSEA to explore the function of their target genes. Although not significantly enriched, *MEPE, PDGFC, MEF2C,* and *VDR* in the *RUNX2* regulon and *PHOSPHO1, SPNS2, COL1A1* in the *CREB3L1* regulon are genes related to skeletal system development in the GO database (Fig. 9A, B). *RUNX2/CREB3L1* regulons were also relatively active in intermediate and mature osteoblasts (Fig. 9C). Expression levels for genes in the *RUNX2* regulon rose gradually during osteoblast maturation, while *CREB3L1* genes were significantly upregulated in mature osteoblasts (Fig. 9D). Intermediate and mature osteoblasts showed high expression levels of *MEPE, PDGFC, MEF2C,* and *VDR* (Fig. 9E) and average expression levels of all *RUNX2* target genes (left plot in 9F). Moreover, high expression levels of *PHOSPHO1, SPNS2,* and *COL1A1* were observed predominantly in mature osteoblasts (Fig. 9E). Average expression levels for all *CREB3L1* target genes rose gradually during osteoblast differentiation, but expression levels increased dramatically in mature osteoblasts (right plot in 9F). Expression of most target genes in the *RUNX2* regulon, including *MEPE, PDGFC, MEF2C,* and *VDR*, increased over pseudotime through the four cell subtype clusters (Fig. 9G)*.* Despite specific target genes of *CREB3L1* displaying a downward trend, genes related to skeletal system development (e.g., *PHOSPHO1, SPNS2* and *COL1A1*) were gradually upregulated during osteoblast maturation (Fig. 9H). The binding motifs for most target genes of *RUNX2* and *CREB3L1* are swissregulon_hs_RUNX1.0.3.p2 and hocomoco_CR3L1_HUMAN.H11MO.0.D, respectively (Fig. 9I, J, Table 1). These results revealed that, as osteoblasts matured, there was an upward trend in *RUNX2*/*CREB3L1* regulons and their target genes related to skeletal development.

### CSN analysis reflected connection changes between target gene pairs

**Fig. 10:**
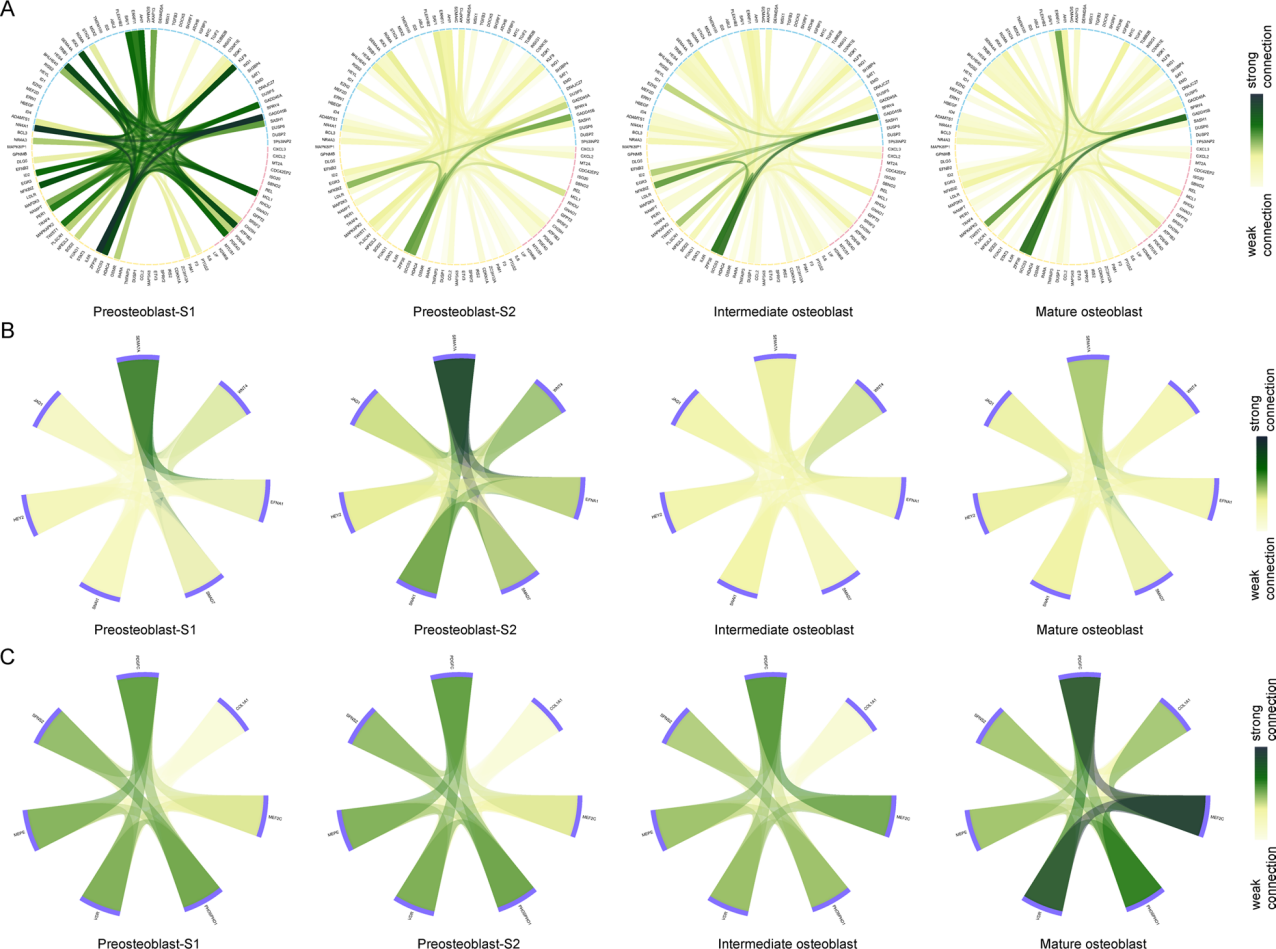
CSN construction among target genes. **A** CSN connections between immunity/cell proliferation/cell differentiation-related target genes in four cell clusters. Red arcs represent immunity-related target genes. Blue arcs represent cell proliferation/cell differentiation-related target genes. Yellow arcs represent immunity and cell proliferation/cell differentiation-related target genes. **B** CSN connections between skeletal system development-related target genes in four cell clusters. Purple arcs represent MSC differentiation-related target genes. **C** CSN connections between skeletal system development-related target genes in four cell clusters. Purple arcs represent skeletal system development-related target genes

Next, we analyzed important target gene connections in the four cell clusters at single-cell resolution. Gene connections in the CSN analysis are the inter-relationships (edges) among gene x and gene y in cell k that are assessed by statistic $\hat{\rho }x\left( k \right)$ (Eq. 1). We used target genes that are related to immunity, cell proliferation/differentiation, mesenchymal stem cell differentiation, and skeletal system development in the *CREM*, *FOSL2*, *FOXC2, RUNX2,* and *CREB3L1* regulons to construct CSN for each cluster (Fig. 10A–C). Strong connections between 113 target genes in the *CREM* and *FOSL2* regulons were established in preosteoblast-S1 (Fig. 10A). Preosteoblasts had the highest connectivity among 7 target genes in the *FOXC2* regulon (Fig. 10B), while mature osteoblasts had the highest connectivity among 7 target genes in the *RUNX2* and *CREB3L1* regulons (Fig. 10C). Thus, immunity, cell proliferation/differentiation, and skeletal system development-related target genes in the *CREM*, *FOSL2*, *RUNX2* and *CREB3L1* regulons were only active in certain cell clusters. Their association network at single-cell resolution also has strong connections in the same cell types; however, strong connections of *FOXC2*’s target genes appeared before (in the early stage) the *FOXC2* regulon attained the highest activation score (during the intermediate stage), which means that target genes in the *FOXC2* regulon might be widely associated with each other in the early cell stage. These results suggest that *FOXC2* may also play a role in the early stage of osteogenic differentiation mediated by target gene interactions.

### Cell trajectory reconstruction reveals a new potential preosteoblast lineage

**Fig. 11:**
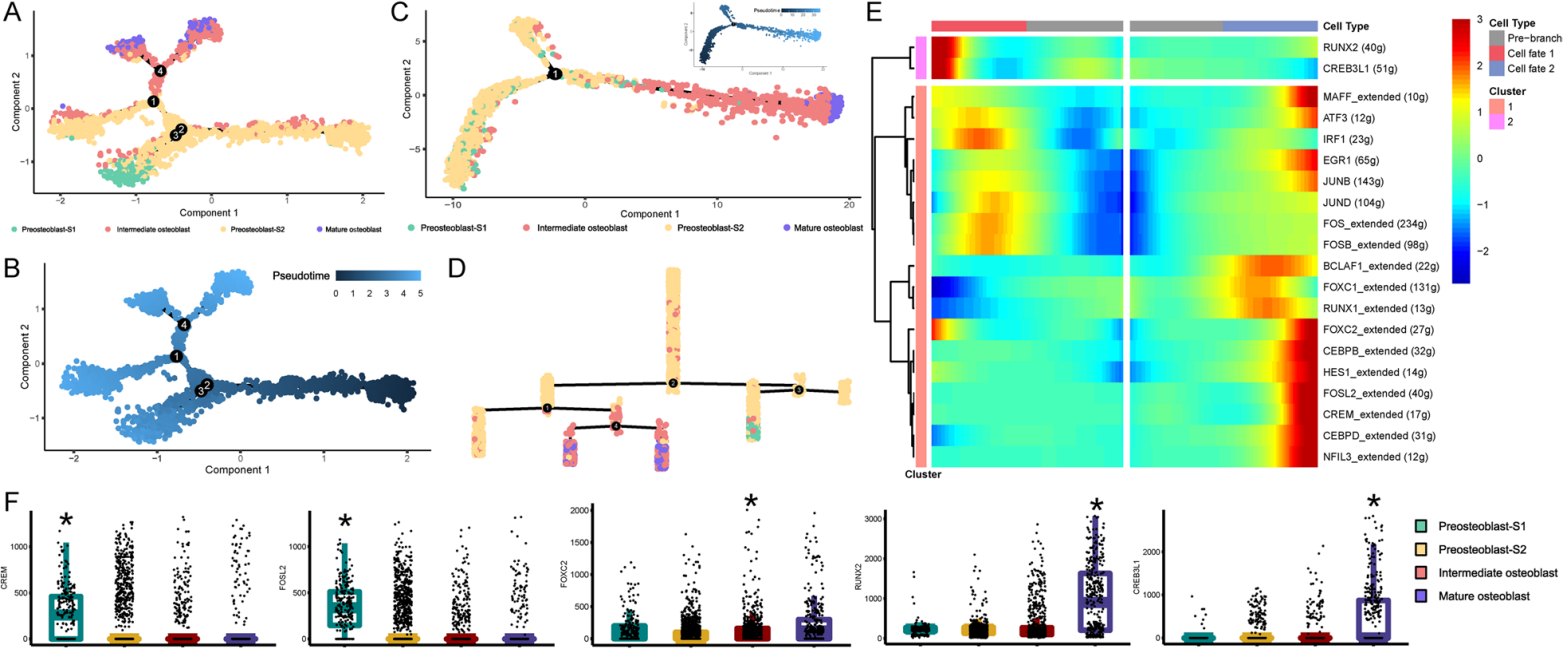
Cell trajectory reconstruction based on regulon activity. **A** Cell developmental trajectory inference based on regulon activity, four branch points in total. **B** The direction of pseudotime in trajectory plot for Fig. 9A. **C** Cell developmental trajectory branch plot based on gene expression. The upper-right trajectory plot indicates the direction of pseudotime. **D** Cell lineage relationships in Fig. 9A. **E** Continuum of dynamic target gene expression around branch point 2 in Fig. 9A, D. Cell fate 1 was correlated to the up branch after branch point 2 in Fig. 9A (left branch after branch point 2 in Fig. 9D). Cell fate 2 was correlated to the down branch after branch point 2 in Fig. 9A (right branch after branch point 2 in Fig. 9D). Pixel color indicates the expression levels. All target genes are clustered in 2 groups based on their expression pattern. **F** NDM of *CREM, FOSL2*, *FOXC2, RUNX2,* and *CREB3L1* TFs. Stars indicate the significance levels of the NDM difference from any other cell clusters (adjusted *p* value < 0.05, Wilcoxon rank-sum test)

To further explore cell lineage from regulon activity aspect, we used regulons activity score matrix to reconstruct the regulon activity score-based cell developmental trajectory (Fig. 11A, B). We found that two preosteoblast subtypes, preosteoblast-S1 and preosteoblast-S2, were highly enriched in early cell stage and mature osteoblast was in the terminal stage (Fig. 11A, B). When we compared these results with the gene expression-based cell trajectory (Fig. 11C), we found that the uptrends of pseudotime values from preosteoblast, intermediate to mature osteoblast were coincident in both regulon score-based (Fig. 11A, B) and gene expression-based (Fig. 11C) cell trajectory results. Additionally, preosteoblast-S1 tended to form a distinct branch in the preosteoblast lineage (Fig. 11A, D), which was quite different in this trajectory structure. Branch heatmap of the branch point 2 in Fig. 11A, D (Fig. 11E) also showed a tendency toward upregulation of *RUNX2* and *CREB3L1* regulons in cell fate 1 (left branch after branch point 2 in Fig. 11D) and upregulation of *CREM* and *FOSL2* regulons in cell fate 2 (right branch after branch point 2 in Fig. 11D). Although several of the 17 active regulons in preosteoblast-S1 showed a different activation tendency, this may be attributed to the mixed-cell composition in such cell fates (Fig. 11E). These results further strengthened the conclusion that preosteoblast-S1 was in a different cell state from the distinct branch of preosteoblast lineage compared with preosteoblast-S2, especially with regard to regulon activity.

To explore gene connections at the whole transcriptome level, we calculated network degrees of five highlighted TFs. *CREM* and *FOSL2* demonstrated the highest NDM values in preosteoblast-S1, *FOXC2* demonstrated the highest NDM values in intermediate osteoblasts, while the NDM values of *RUNX2* and *CREB3L1* approached their peaks in mature osteoblasts (Fig. 11F). These results indicate that more interactions exist between these TFs and other genes in the corresponding cell subtypes. These are also the same trends identified for the activity of the corresponding regulons.

**Fig. 12:**
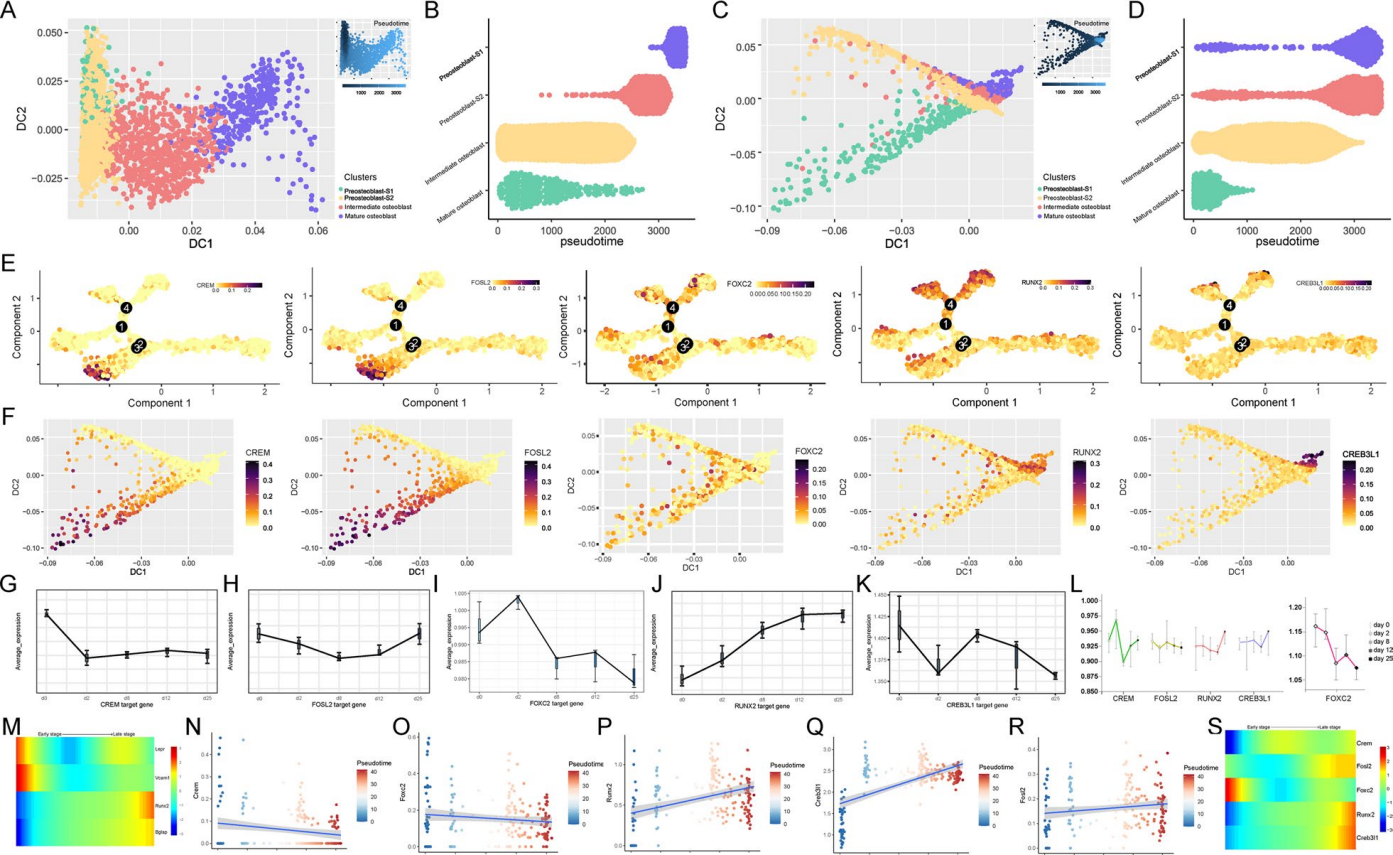
Validation of regulon activity tendency. **A** Diffusion map trajectory inference based on gene expression. The upper-right trajectory plot indicates the direction of pseudotime. **B** Cell distribution based on the pseudotime coordinates (Fig. 10A). The *x*-axis is the pseudotime and the *y*-axis represents the osteoblast subtypes. **C** Diffusion map trajectory inference based on regulon activity. The upper-right trajectory plot indicates the direction of pseudotime. **D** Cell distribution based on the pseudotime coordinates in (Fig. 10C). The *x*-axis is pseudotime and the *y*-axis represents osteoblast subtypes. **E** Monocle trajectory heatmap of *CREM, FOSL2*, *FOXC2, RUNX2,* and *CREB3L1* regulons. **F** Diffusion map trajectory heatmap of *CREM, FOSL2*, *FOXC2, RUNX2,* and *CREB3L1* regulons. **G** Immunity and cell proliferation/differentiation-related target genes’ average expression in *CREM* regulon. **H** Immunity and cell proliferation/differentiation-related target genes’ average expression in *FOSL2* regulon. **I** MSC differentiation-related target genes’ average expression in *FOXC2* regulon. **J** Average expression of all target genes in *RUNX2* regulon. **K** Skeletal system development-related target genes’ average expression in *CREB3L1* regulon. **L** Expression of *CREM, FOSL2*, *FOXC2, RUNX2,* and *CREB3L1* genes during osteogenic differentiation process in vitro. **M** Pseudotime heatmap of *Lepr*, *Vcam1*, *Runx2* and *Bglap* during osteogenic differentiation in vivo on mouse. Pixel color indicates expression levels. **N** Immunity and cell proliferation/differentiation-related target genes’ average expression in *Crem* regulon. **O** Immunity and cell proliferation/differentiation-related target genes’ average expression in *Fosl2* regulon. **P** MSC differentiation-related target genes’ average expression in *Foxc2* regulon. **Q** Skeletal system development-related target genes’ average expression in *Runx2* regulon. **R** Skeletal system development-related target genes’ average expression in *Creb3l1* regulon. **S** Pseudotime heatmap of *Crem*, *Fosl2*, *Foxc2, Runx2,* and *Creb3l1* genes during osteogenic differentiation in vivo on mouse. Pixel color indicates the expression levels

We also used diffusion mapping [28] to further confirm our osteogenic differentiation trajectory results based on gene expression and TF regulation aspects. Pseudotime order of preosteoblasts (preosteoblast-S1, preosteoblast-S2), intermediate osteoblasts, and mature osteoblasts in diffusion map analysis results were consistent with our earlier Monocle-based analysis (Fig. 12A–D). Compared with preosteoblast-S2, diffusion map analysis showed that preosteoblast-S1 cells were more concentrated in the DC2 dimension (Fig. 12A) and the early stage of pseudotime (< 1500, Fig. 12B) in gene expression-based trajectory. Preosteoblast-S1 also tended to form a distinctive branch in preosteoblast lineage in TF regulation-based trajectory (Fig. 12C, D). Thus, preosteoblast-S1 performed differently than preosteoblast-S2, even with the independent approach using gene expression-based “traditional” pseudotime analysis. These differences were more apparent in TF regulation-based trajectories, which was consistent with the results from the Monocle analysis. These results further validated the independence of preosteoblast-S1. *CREM, FOSL2, FOXC2, RUNX2,* and *CREB3L1* regulon coincident active tendencies were also confirmed in monocle (Fig. 12E) and diffusion map trajectory heatmap (Fig. 12F). Thus, *CREM* and *FOSL2* regulons were highly active in the early cell stage (preosteoblast-S1), *FOXC2* regulon was highly active in the intermediate cell stage, and *RUNX2* and *CREB3L1* regulons were highly active in the late cell stage (mature osteoblast).

### Validation of regulon expression in vitro and in vivo

One major limitation of these results is that they were all generated with cells recovered from one patient. To validate our results, we analyzed expression levels of target genes for the *CREM, FOSL2, FOXC2, RUNX2,* and *CREB3L1* regulons during osteogenic differentiation of BM-MSCs to osteoblasts in vitro.

We obtained data for osteogenic differentiation in vitro from the GEO database with accession number GSE37558 [30]. Average expression of immunity and cell proliferation/differentiation-related target genes in the *CREM* and *FOSL2* regulons were higher in the early stage (day 0), except for the *FOSL2* regulon which was elevated again on day 25 (Fig. 12G, H). Average expression of mesenchymal cell differentiation-related target genes in the *FOXC2* regulons were higher on day 2 (Fig. 12I). Average expression for all target genes in the *RUNX2* regulon showed an upward tendency during osteogenic differentiation (from day 0 to day 25, Fig. 12J); however, expression levels for target genes of the *CREB3L1* regulon showed somewhat erratic changes (Fig. 12K). Expression levels of *CREM* and *FOSL2* genes were also highest in the early stages (day 0/day 2, Fig. 12L). *RUNX2* and *CREB3L1* genes approached their peaks in later stages (day 25), as expected (Fig. 12L), while TF *FOXC2’*s expression was earlier than expected (early stages, day 0/day 2, Fig. 12L). Despite the limitations of in vitro conditions, and limited temporal sampling and sample size, results from these two markedly different experimental conditions are highly consistent. Thus, both TF and target gene expression tendencies in the five proposed regulons (*CREM, FOSL2, FOXC2, RUNX2,* and *CREB3L1*) were confirmed by independent analysis of this in vitro data for osteogenic differentiation (Fig. 12G–L), providing valuable validation of our scRNA-seq based analysis.

To further validate our osteogenic differentiation pseudotime results, we analyzed another in vivo scRNA-seq dataset using mouse osteoblasts, acquired from the GEO database under the accession number GSE108891 [31]. During osteogenesis in mice, the expression of BM-MSC-related markers *Lepr* [33] and *Vcam1* [34] gradually decreased over pseudotime. Further, the expression of osteoblast-related markers *Runx2* [8] and *Bglap* [35] gradually increased as osteogenesis progressed (Fig. 12M). These trends were consistent with results from our human scRNA-seq data analysis (Fig. 2C). Additionally, the downward tendency of target gene expression in *Crem* regulons (Fig. 12N), downward tendency of TF and target gene expression in *Foxc2* regulon in late cell stage (Fig. 12O), and upward tendency of TF and target gene expression in *Runx2* and *Creb3l1* regulons (Fig. 12P, Q) were confirmed by this in vivo data using mouse osteoblasts. The inconsistent tendency of no obvious decrease in expression of target genes of the *Fosl2* regulon (Fig. 12R), and TF expression in *Crem* and *Fosl2* regulons (Fig. 12S), may be attributable to species differences, suggesting the necessity and importance of using human specimens to study osteogenesis. These in vivo results using mouse osteoblasts further validate our pseudotime-based cell cluster designations of preosteoblasts, intermediate osteoblasts, and mature osteoblasts during human osteogenesis.

**Fig. 13:**
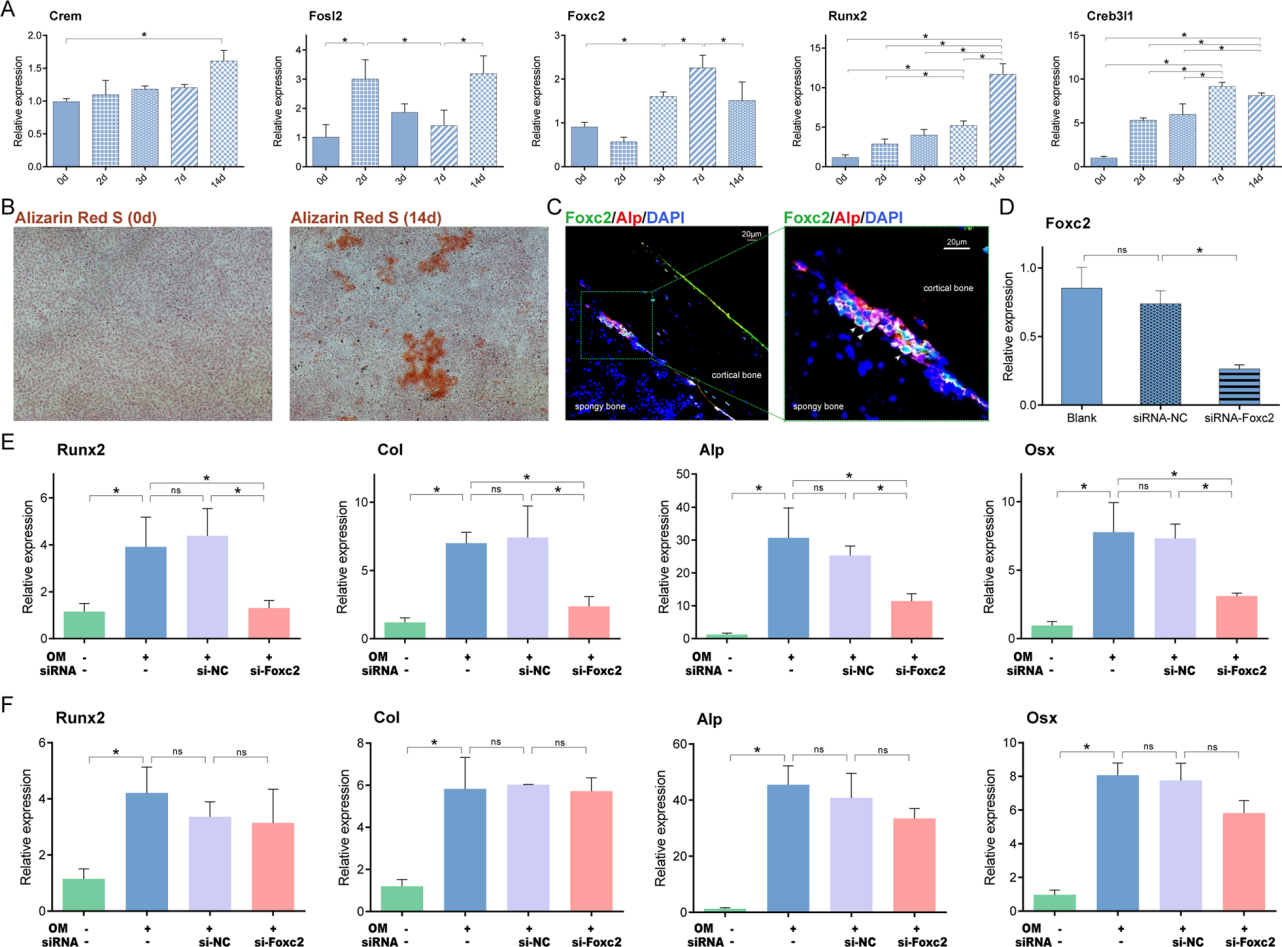
Experimental validation in MC3T3-E1 preosteoblastic cell line. **A** Expression of *Crem, Fosl2*, *Foxc2*, *Runx2* and *Creb3l1* genes during osteogenic differentiation process in 0, 2, 3, 7 and 14 days. **B** Alizarin Red S staining at 0 and 14 days. **C** Representative confocal immunofluorescent imaging showing distribution of Foxc2^+^Alp^+^ osteoblast in murine femur. Arrows mark Foxc2^+^Alp^+^ cells. **D** Qpcr experiment revealed the successful knockdown of Foxc2. **E** Gene expression of *Runx2, Col, Alp* and *Osx* at 3 days. **F** Gene expression of *Runx2, Col, Alp* and *Osx* at 7 days. (*, *P* < 0.05 by one-way ANOVA; *ns* not significant, *OM* osteogenic differentiation medium)

Finally, we further validated our osteogenic differentiation results using the mouse preosteoblast cell line MC3T3-E1. During the osteogenic induction process, *Crem*’s upregulation was later than expected at 14 days. *Fosl2* had the highest level at 2 days, then downregulated at 3 and 7 days, which was consistent with its early-stage activation tendency. However, instead of staying downregulated as we predicted, *Fosl2*’s expression elevated again at day 14 which, may be attributable to species differences and in vitro conditions. *Foxc2* was upregulated at 3 and 7 days (intermediate stage) and began to decrease at 14 days as expected. *Runx2* and *Creb3l1* genes reached their peaks at later stages (7 and 14 days), as expected (Fig. 13A). The success of osteogenesis induction was demonstrated by increased mineral nodule formation at 14 days in Alizarin red S staining results (Fig. 13B).

### *Foxc2* is critical to osteogenesis process in MC3T3-E1 cells

*Foxc2*’s function in the osteogenesis process is unclear. *Foxc2* gene's upregulation at the intermediate stage was concordant in the scRNA-seq analysis and osteogenesis induction experiment results; however, our CSN results also showed its potential role in early stage of differentiation. Therefore, we further explored *Foxc2*’s function in vivo and in vitro. First, immunofluorescence on mouse femur displayed the co-staining of the osteoblast marker *Alp* with *Foxc2*, thereby verifying the expression of *Foxc2* in osteoblasts in vivo (Fig. 13C). To further explore the biological function of *Foxc2* in osteogenic differentiation, we performed *Foxc2* siRNA knockdown experiment on MC3T3-E1 cells. Successful knockdown of *Foxc2* was verified by qPCR analysis (Fig. 13D). Silencing of Foxc2 at 0–3 days (early stage) decreased osteoblastic differentiation marker gene expression including *Runx2, Col, Alp,* and *Osx* (Fig. 13E), whereas silencing of *Foxc2* at 4–7 days (intermediate stage) did not affect osteoblastic differentiation (Fig. 13F). These results suggest that *Foxc2* plays an important role in osteogenic differentiation at the early stage.

## Discussion

In the current study, we performed TF network and CSN analysis, at the single-cell level, in human osteoblasts freshly isolated from the femur of a 31-year-old man who underwent hip replacement surgery.

Our work revealed five important regulons, *CREM*, *FOSL2, FOXC2, RUNX2,* and *CREB3L1,* whose varying activity levels enabled us to identify four distinct cell clusters that we designated preosteoblast-S1, preosteoblast-S2, intermediate osteoblast, and mature osteoblast, representing different maturational stages during human osteogenesis. *CREM* and *FOSL2* were most active in preosteoblasts, *FOXC2* was most active in intermediate osteoblasts, whereas *RUNX2* and *CREB3L1* activity increased as osteoblasts matured. Since these results were based on single-cell analysis of a single human subject, we validated our results using two different approaches. Comparable results were generated using human osteoblasts cultured in vitro and an in vivo scRNA-seq dataset for mouse osteoblasts.

These findings provide a framework for gene relationships during osteogenesis at the single-cell level, laying the foundation for exploring characteristic gene functions from a novel TF regulation perspective. Linking genomic regulatory patterns to variations in gene expression at the single-cell level could be robust against drop-out commonly seen in single-cell sequencing data, thereby optimizing the discovery and characterization of cellular states [20]. Additionally, unlike traditional network construction groups of cells, the CSN method constructs separate networks at the single-cell level, thus the heterogeneity of each individual cell is preserved [16, 19]. To this end, our research revealed unique features in the regulon activity landscape and reciprocal gene interactions within each human osteoblast subtype. Inferred TF and target genes in those candidate functional regulons may provide valuable clues for subsequent pathophysiological study of osteoblast metabolism and osteoarthritis.

Cell heterogeneity may manifest in a variety of different ways (e.g., gene expression or TF regulation). Consequently, application of related conjoint analysis (using Seurat and SCENIC) is important in providing more comprehensive results in cell type identification. Activity of the regulons we studied differed substantially among the four designated cell clusters. Regulon activity-based cell clustering and trajectory inference effectively supplement the traditional approach results that preosteoblast-S1 was re-identified as an independent preosteoblast subtype in a distinctive cell lineage branch; meanwhile, the pseudotime order of intermediate osteoblasts and mature osteoblasts also obtained further validation. Although some cell lineage branches need further exploration and validation, our results showed that preosteoblast-S1 represents a potential independent state of cellular differentiation, and mature osteoblasts represent the final cell fate in terms of both gene expression and TF regulation. GSEA results further confirmed the immunity/cell proliferation/cell differentiation and skeletal system developmental functions of these two cell types.

*CREM* and *FOSL2* regulons were relatively active in preosteoblast-S1. Strong CSN connections for immunity and cell proliferation/differentiation-related target genes in *CREM* and *FOSL2* regulons further supported their designated functional states. *CREM* has been reported to be a member of the ATF/CREB family of basic leucine zipper transcription factors [36, 37]. *CREM* encodes a variety of different isoforms by utilizing four promoters and a complex pattern of alternative mRNA splicing [38]. For example, ICER is a dominant negative transcription regulator transcribed by the P2 promoter of the *CREM* gene. Osteoblast-targeted overexpression of ICER resulted in osteopenia, attributable primarily to reduced bone formation [39]. Our findings demonstrate that the *CREM* regulon was most active in preosteoblast-S1. Thus, the combined action of various *CREM* isoforms, which may impact specific bone formation and homeostasis regulation processes in this cell subtype, warrants further in-depth research. The *FOSL2* regulon was another relatively active regulon in preosteoblast-S1. *FOSL2* is a paradigm transcription factor that controls the endocrine function of skeletal system. *FOSL2* expression in osteoblasts influences adiponectin and osteocalcin expression and affects metabolism [36, 37, 40]. Activation patterns of the *FOSL2* regulon might indicate that skeleton-endocrine functions of *FOSL2* are performed predominantly by preosteoblast-S1. Previous studies showed that interactions of *FOSL2* and proinflammatory cytokines, including *IL-6,* contributed to various inflammatory reactions [41–43]. In addition, *FOSL2* regulon’s target gene, *IL-6,* was included in the core gene modules of immunity-related PPI networks. As inflammatory activity is critical to the pathogenesis of osteoarthritis [44], determining whether the *FOSL2* regulon contributed to osteoarthritis-associated inflammation via *IL-6,* or other immunity-related genes, warrants further study.

We further showed that the highly active regulon *MXD4* [45], *KLF2* [46] in preosteoblasts S2 and *TAF7* [47] in intermediate osteoblasts were related to the osteogenesis process. However, limited research [48] reported *FOXC2’s* osteogenesis function in preosteoblasts, especially at the early and intermediate stages. After verifying the expression of *Foxc2* in osteoblasts in vivo, our knockdown experiments by siRNA further suggested that *Foxc2* mainly influenced the osteogenic differentiation process in the early stage. These results were concordant with our CSN results, and as this stage was also the fastest increasing stage for *Foxc2* gene expression and regulon activity, results indicate the importance of using multiple analysis approaches in scRNA-seq data interpretation.

*RUNX2* is a classical osteogenic-related TF which is essential for osteoblast differentiation and maturation [49–51]. We further confirmed that *RUNX2* regulon activity and expression of its target genes were elevated in late-stage osteoblasts. Endoplasmic reticulum stress transducer old astrocyte specifically induced substance (OASIS), encoded by *CREB3L1*, has been demonstrated to promote the terminal maturation of osteoblasts [52–54]. OASIS activates the transcription of type I collagen gene *COL1A1* and affects the secretion of bone matrix proteins [55–57]. These previous studies support our results demonstrating *CREB3L1* regulon’s active tendency, the trend of *COL1A1*’s CSN connection, and the *COL1A1*-*CREB3L1* regulation edge. In addition, to investigate the gene association of *CREM, FOSL2, FOXC2, RUNX2,* and *CREB3L1* at the whole transcriptome level, we calculated NDM values of these five TFs. As expected, NDM values of *CREM* and *FOSL2* were highest in preosteoblast-S1, NDM values of *FOXC2* were highest in intermediate osteoblasts, and NDM values of *RUNX2* and *CREB3L1* were highest in mature osteoblasts*.* These results demonstrate the wide influence of these regulons at the whole transcriptome level by directly or indirectly regulating gene targets, co-expression, alternative splicing, or other potential mechanisms [16].

There are several limitations to the present study. Most significantly, much of the research data were obtained from one 31-year-old Chinese male patient with osteoarthritis and osteopenia. Independent in vitro data and complementary analysis with diffusion mapping provided further evidence supporting the differential TF expression and pattern dynamics of the regulons identified in cells from this patient. Nevertheless, this sample size is limited in its ability to represent the general population with respect to bone health or disorder. Consequently, the cell subtype designations reported in the current study must be considered preliminary until we have an opportunity to explore physiological differences of these cell subtypes from a larger number, and greater diversity, of subjects. Specifically, more samples from both healthy subjects and bone disorder patients are needed to derive unbiased expression matrices for downstream analyses of regulons and gene interactions in osteoblasts as they differentiate. Another limitation is that the interactions in the PPI network are inferred rather than assured in the cells under study, since the PPIs from STRING are not tissue-specific. Although evidence of interaction like correlation/regulation relationships and protein-binding validated in Co-IP or other in vitro experiments also lends support to the potential interactions in the cells under study [58–60], further experimental validation should be performed with a sufficient sample size in vivo to validate the results from the regulon network and PPI analyses.

## Conclusion

Despite these potential limitations, our results provide the first necessary and valuable insights into the cellular heterogeneity of osteoblasts, along with a comprehensive and systematic understanding of cell development and functional state changes of primary osteoblasts. These insights, based on both TF regulation and CSN perspectives at the single-cell network level, may prove critical to understanding bone metabolism and pathophysiologic mechanisms associated with various bone disorders. Multiple functions like immunity, endocrine activity, cell differentiation, and cell development were differentially influenced by different TFs in different cell stages. Our work also provides a new approach of integrating analysis for novel CSN methods with classical TF regulatory network in scRNA-seq data. The findings provide crucial insights from a novel regulatory network perspective that warrant further exploration in functional mechanistic studies in bone physiological and pathological processes.

## Supplementary Information

**Additional file 1**. **Fig. S1. Additional materials for scRNA-seq data analysis**. A. Number of genes per cell. Blue lines represent the limit values for quality control. B. Elbow plot of stdev for top 18 PCs. C. The percentage of variance associated with top 18 PCs. Line chart shows the percentage of variance associated with each individual PC. Cumulative percentage of variance were showed in each bar.

**Additional file 2**. **Fig. S2. Additional materials for GSEA analysis**. A. Other cell proliferation/differentiation-related GSEA analysis results in target genes of active regulons in preosteoblast-S1. B. Other immunity-related GSEA analysis results in target genes of active regulons in preosteoblast-S1.

## Data Availability

The scRNA-seq data for primary osteoblasts from human sample can be accessed with accession number under GSE147390 in GEO database. The gene expression profile of osteogenic differentiation process from human BM-MSCs to osteoblasts in vitro was obtained from the GEO database with accession numbers GSE37558. The scRNA-seq dataset in vivo on mouse osteoblasts which was acquired from GEO database under the accession numbers of GSE108891.

## Abbreviations

TF: Transcription factor
SCENIC: Single-cell regulatory network inference and clustering
CSN: Cell-specific network
RUNX2: Runt-related transcription factor 2
scRNA-seq: Single-cell RNA sequencing
BMD: Bone mineral density
PCA: Principal component analysis
PCs: Principal components
UMAP: Uniform manifold approximation and projection
AUC: Area under the curve
GSEA: Gene set enrichment analysis
NES: Normalized enrichment score
PPI: Protein–protein interaction
STRING: Search Tool for the Retrieval of Interacting Genes
MCODE: Molecular complex detection
NDM: Network degree matrix
BM-MSCs: Bone marrow-derived mesenchymal stem cells
NC: Negative control
LEPR: Leptin receptor
VCAM1: Vascular cell adhesion molecule 1
OASIS: Old astrocyte specifically induced substance

## References

[1] Al-Bari A, Al MA (2020). Current advances in regulation of bone homeostasis. FASEB BioAdv.

[2] Civitelli R (2008). Cell-cell communication in the osteoblast/osteocyte lineage. Arch Biochem Biophys.

[3] Friedman M, Oyserman S, Hankenson K (2009). Wnt11 promotes osteoblast maturation and mineralization through R-spondin 2. J Biol Chem.

[4] Nurminskaya M, Magee C, Faverman L, Linsenmayer T (2003). Chondrocyte-derived transglutaminase promotes maturation of preosteoblasts in periosteal bone. Dev Biol.

[5] Iacobini C, Fantauzzi C, Pugliese G, Menini S (2017). Role of galectin-3 in Bone cell differentiation, bone pathophysiology and vascular osteogenesis. Int J Mol Sci.

[6] Valcourt U, Gouttenoire J, Moustakas A, Herbage D, Mallein-Gerin F (2002). Functions of transforming growth factor-beta family type I receptors and Smad proteins in the hypertrophic maturation and osteoblastic differentiation of chondrocytes. J Biol Chem.

[7] Nakashima K, de Crombrugghe B (2003). Transcriptional mechanisms in osteoblast differentiation and bone formation. Trends Genet.

[8] Schroeder T, Jensen E, Westendorf J (2005). Runx2: a master organizer of gene transcription in developing and maturing osteoblasts. Birth Defects Res C Embryo Today.

[9] Meyer M, Benkusky N, Pike J (2014). The RUNX2 cistrome in osteoblasts: characterization, down-regulation following differentiation, and relationship to gene expression. J Biol Chem.

[10] Gomathi K, Akshaya N, Srinaath N, Moorthi A, Selvamurugan N (2020). Regulation of Runx2 by post-translational modifications in osteoblast differentiation. Life Sci.

[11] Komori T (2022). Whole aspect of Runx2 functions in skeletal development. Int J Mol Sci.

[12] Liu T, Lee E (2013). Transcriptional regulatory cascades in Runx2-dependent bone development. Tissue Eng Part B Rev.

[13] Guenther H, Hofstetter W, Stutzer A, Mühlbauer R, Fleisch H (1989). Evidence for heterogeneity of the osteoblastic phenotype determined with clonal rat bone cells established from transforming growth factor-beta-induced cell colonies grown anchorage independently in semisolid medium. Endocrinology.

[14] Liu F, Malaval L, Aubin J (1997). The mature osteoblast phenotype is characterized by extensive plasticity. Exp Cell Res.

[15] Gong Y, Yang J, Li X, Zhou C, Chen Y, Wang Z (2021). A systematic dissection of human primary osteoblasts at single-cell resolution. Aging.

[16] Dai H, Li L, Zeng T, Chen L (2019). Cell-specific network constructed by single-cell RNA sequencing data. Nucleic Acids Res.

[17] Wang S, Zheng Y, Li J, Yu Y, Zhang W, Song M (2020). Single-cell transcriptomic atlas of primate ovarian aging. Cell.

[18] Hao Q, Li J, Zhang Q, Xu F, Xie B, Lu H (2021). Single-cell transcriptomes reveal heterogeneity of high-grade serous ovarian carcinoma. Clin Transl Med.

[19] Wang S, Gong Y, Wang Z, Greenbaum J, Xiao H, Deng H (2021). Cell-specific network analysis of human folliculogenesis reveals network rewiring in antral stage oocytes. J Cell Mol Med.

[20] Aibar S, González-Blas C, Moerman T, Huynh-Thu V, Imrichova H, Hulselmans G (2017). SCENIC: single-cell regulatory network inference and clustering. Nat Methods.

[21] Hou X, Tian F (2022). STAT3-mediated osteogenesis and osteoclastogenesis in osteoporosis. Cell Commun Signal.

[22] Liu Q, Li M, Wang S, Xiao Z, Xiong Y, Wang G (2020). Recent advances of osterix transcription factor in osteoblast differentiation and bone formation. Front Cell Dev Biol.

[23] Almeida M, Porter R (2019). Sirtuins and FoxOs in osteoporosis and osteoarthritis. Bone.

[24] Zhong S, Ding W, Sun L, Lu Y, Dong H, Fan X (2020). Decoding the development of the human hippocampus. Nature.

[25] Stuart T, Butler A, Hoffman P, Hafemeister C, Papalexi E, Mauck W (2019). Comprehensive integration of single-cell data. Cell.

[26] Butler A, Hoffman P, Smibert P, Papalexi E, Satija R (2018). Integrating single-cell transcriptomic data across different conditions, technologies, and species. Nat Biotechnol.

[27] Trapnell C, Cacchiarelli D, Grimsby J, Pokharel P, Li S, Morse M (2014). The dynamics and regulators of cell fate decisions are revealed by pseudotemporal ordering of single cells. Nat Biotechnol.

[28] Angerer P, Haghverdi L, Büttner M, Theis F, Marr C, Buettner F (2016). *destiny*: diffusion maps for large-scale single-cell data in R. Bioinform (Oxf Engl).

[29] Bader GD, Hogue CWV (2003). An automated method for finding molecular complexes in large protein interaction networks. BMC Bioinform.

[30] Alves R, Eijken M, van de Peppel J, van Leeuwen J (2014). Calcifying vascular smooth muscle cells and osteoblasts: independent cell types exhibiting extracellular matrix and biomineralization-related mimicries. BMC Genom.

[31] Tikhonova A, Dolgalev I, Hu H, Sivaraj K, Hoxha E, Cuesta-Domínguez Á (2019). The bone marrow microenvironment at single-cell resolution. Nature.

[32] Szklarczyk D, Gable A, Nastou K, Lyon D, Kirsch R, Pyysalo S (2021). The STRING database in 2021: customizable protein-protein networks, and functional characterization of user-uploaded gene/measurement sets. Nucleic Acids Res.

[33] Zhou B, Yue R, Murphy M, Peyer J, Morrison S (2014). Leptin-receptor-expressing mesenchymal stromal cells represent the main source of bone formed by adult bone marrow. Cell Stem Cell.

[34] Akiyama K, You Y, Yamaza T, Chen C, Tang L, Jin Y (2012). Characterization of bone marrow derived mesenchymal stem cells in suspension. Stem Cell Res Ther.

[35] Itoh Y, Itoh S, Naruse H, Kagioka T, Hue M, Abe M (2021). Intracellular density is a novel indicator of differentiation stages of murine osteoblast lineage cells. J Cell Biochem.

[36] Xie J, Deng Z, Alahdal M, Liu J, Zhao Z, Chen X (2021). Screening and verification of hub genes involved in osteoarthritis using bioinformatics. Exp Ther Med.

[37] Bozec A, Bakiri L, Jimenez M, Rosen E, Catalá-Lehnen P, Schinke T (2013). Osteoblast-specific expression of Fra-2/AP-1 controls adiponectin and osteocalcin expression and affects metabolism. J Cell Sci.

[38] Liu F, Huang Y, Kream B (2005). Identification of novel cAMP responsive element modulator (CREM) isoforms expressed by osteoblasts. Calcif Tissue Int.

[39] Chandhoke T, Huang Y, Liu F, Gronowicz G, Adams D, Harrison J (2008). Osteopenia in transgenic mice with osteoblast-targeted expression of the inducible cAMP early repressor. Bone.

[40] Bozec A, Bakiri L, Jimenez M, Schinke T, Amling M, Wagner E (2010). Fra-2/AP-1 controls bone formation by regulating osteoblast differentiation and collagen production. J Cell Biol.

[41] Chen L, Cheng S, Sun K, Wang J, Liu X, Zhao Y (2021). Changes in macrophage and inflammatory cytokine expressions during fracture healing in an ovariectomized mice model. BMC Musculoskelet Disord.

[42] Huang L, Wu H, Wu Y, Song F, Zhang L, Li Z, et al. Pcsk9 knockout aggravated experimental apical periodontitis via LDLR. J Dent Res. 2021:220345211015128. 10.1177/00220345211015128 34036816

[43] Zhang Q, Song X, Chen X, Jiang R, Peng K, Tang X (2021). Antiosteoporotic effect of hesperidin against ovariectomy-induced osteoporosis in rats via reduction of oxidative stress and inflammation. J Biochem Mol Toxicol.

[44] Montero C, Riquelme G, Campo M, Lagos N (2021). Neosaxitoxin, a Paralytic Shellfish Poison phycotoxin, blocks pain and inflammation in equine osteoarthritis. Toxicon Off J Int Soc Toxinol..

[45] Hoogendam J, Farih-Sips H, van Beek E, Löwik C, Wit J, Karperien M (2007). Novel late response genes of PTHrP in chondrocytes. Horm Res.

[46] Kim I, Kim J, Kim K, Seong S, Kim N (2019). The IRF2BP2-KLF2 axis regulates osteoclast and osteoblast differentiation. BMB Rep.

[47] Bao S, Guo Y, Diao Z, Guo W, Liu W (2020). Genome-wide identification of lncRNAs and mRNAs differentially expressed in human vascular smooth muscle cells stimulated by high phosphorus. Ren Fail.

[48] You W, Gao H, Fan L, Duan D, Wang C, Wang K (2013). Foxc2 regulates osteogenesis and angiogenesis of bone marrow mesenchymal stem cells. BMC Musculoskelet Disord.

[49] Guasto A, Cormier-Daire V (2021). Signaling pathways in bone development and their related skeletal dysplasia. Int J Mol Sci.

[50] Montecino M, Carrasco M, Nardocci G (2020). Epigenetic control of osteogenic lineage commitment. Front Cell Dev Biol.

[51] Zhang J, Pan J, Jing W (2020). Motivating role of type H vessels in bone regeneration. Cell Prolif.

[52] Guillemyn B, Kayserili H, Demuynck L, Sips P, De Paepe A, Syx D (2019). A homozygous pathogenic missense variant broadens the phenotypic and mutational spectrum of CREB3L1-related osteogenesis imperfecta. Hum Mol Genet.

[53] Asada R, Saito A, Kawasaki N, Kanemoto S, Iwamoto H, Oki M (2012). The endoplasmic reticulum stress transducer OASIS is involved in the terminal differentiation of goblet cells in the large intestine. J Biol Chem.

[54] Yu S, Guo J, Sun Z, Lin C, Tao H, Zhang Q (2021). BMP2-dependent gene regulatory network analysis reveals Klf4 as a novel transcription factor of osteoblast differentiation. Cell Death Dis.

[55] Sekiya H, Murakami T, Saito A, Hino S, Tsumagari K, Ochiai K (2010). Effects of the bisphosphonate risedronate on osteopenia in OASIS-deficient mice. J Bone Miner Metab.

[56] Yumimoto K, Matsumoto M, Onoyama I, Imaizumi K, Nakayama K (2013). F-box and WD repeat domain-containing-7 (Fbxw7) protein targets endoplasmic reticulum-anchored osteogenic and chondrogenic transcriptional factors for degradation. J Biol Chem.

[57] Murakami T, Hino S, Nishimura R, Yoneda T, Wanaka A, Imaizumi K (2011). Distinct mechanisms are responsible for osteopenia and growth retardation in OASIS-deficient mice. Bone.

[58] Lu Y, Li K, Hu Y, Wang X (2021). Expression of immune related genes and possible regulatory mechanisms in Alzheimer's disease. Front Immunol.

[59] Liu Y, Dong Y, Wu X, Wang X, Niu J (2022). Identification of immune microenvironment changes and the expression of immune-related genes in liver cirrhosis. Front Immunol.

[60] Zhang Y, Li W, Zhou Y (2020). Identification of hub genes in diabetic kidney disease via multiple-microarray analysis. Ann Transl Med.

