# Supplementary figures and images for: Regulon active landscape reveals cell development and functional state changes of human primary osteoblasts in vivo
Source: Hum Genomics. 2023 Feb 15;17:11. doi: 10.1186/s40246-022-00448-2 (PMC9930257; doi:10.1186/s40246-022-00448-2)

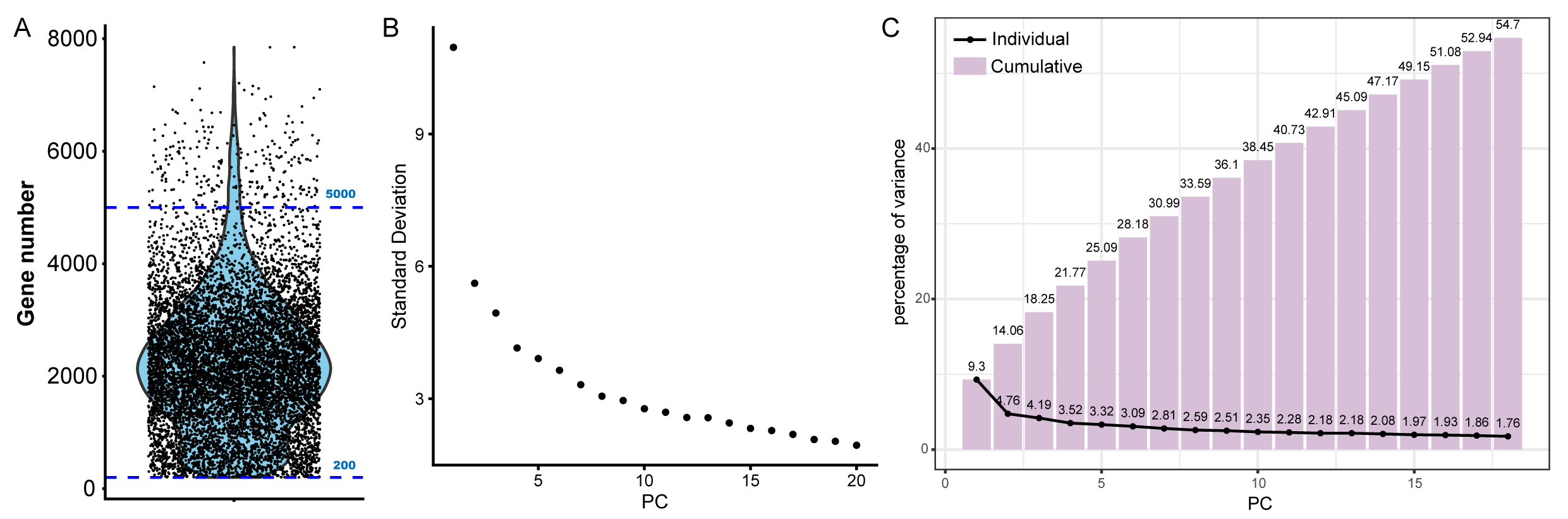

Supplement: Supplementary file 1 — Additional file 1. Fig. S1. Additional materials for scRNA-seq data analysis. A. Number of genes per cell. Blue lines represent the limit values for quality control. B. Elbow plot of stdev for top 18 PCs. C. The percentage of variance associated with top 18 PCs. Line chart shows the percentage of variance associated with each individual PC. Cumulative percentage of variance were showed in each bar. [file 40246_2022_448_MOESM1_ESM.tif]

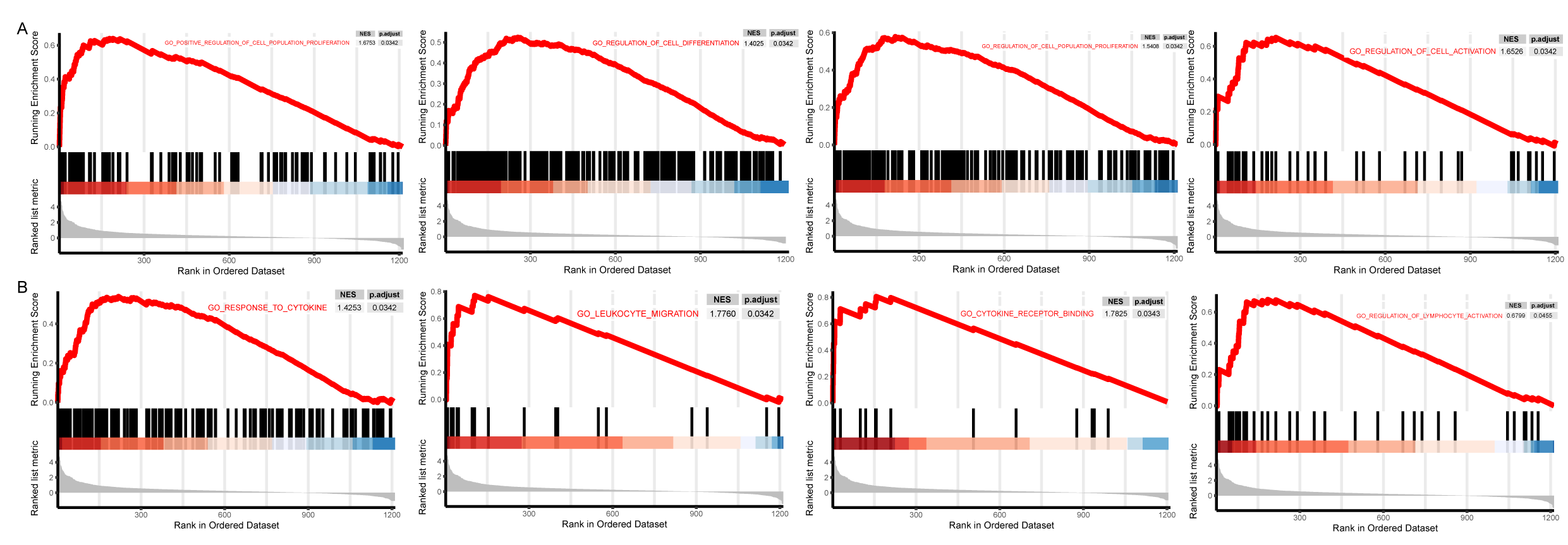

Supplement: Supplementary file 2 — Additional file 2. Fig. S2. Additional materials for GSEA analysis. A. Other cell proliferation/differentiation-related GSEA analysis results in target genes of active regulons in preosteoblast-S1. B. Other immunity-related GSEA analysis results in target genes of active regulons in preosteoblast-S1. [file 40246_2022_448_MOESM2_ESM.tif]
